# Programming Molecular Systems To Emulate a Learning Spiking Neuron

**DOI:** 10.1021/acssynbio.1c00625

**Published:** 2022-05-27

**Authors:** Jakub Fil, Neil Dalchau, Dominique Chu

**Affiliations:** † APT Group, School of Computer Science, The University of Manchester, Manchester M13 9PL, United Kingdom; ‡ Microsoft Research, Cambridge CB1 2FB, United Kingdom; § CEMS, School of Computing, University of Kent, Canterbury CT2 7NF, United Kingdom

**Keywords:** Hebbian learning, spiking neurons, DNA strand displacement, autonomous learning, biochemical intelligence

## Abstract

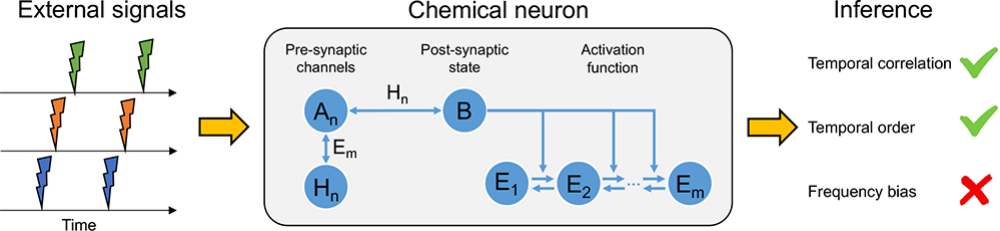

Hebbian theory seeks to explain how the neurons in the brain adapt to stimuli to enable learning. An interesting feature of Hebbian learning is that it is an unsupervised method and, as such, does not require feedback, making it suitable in contexts where systems have to learn autonomously. This paper explores how molecular systems can be designed to show such protointelligent behaviors and proposes the first chemical reaction network (CRN) that can exhibit autonomous Hebbian learning across arbitrarily many input channels. The system emulates a spiking neuron, and we demonstrate that it can learn statistical biases of incoming inputs. The basic CRN is a minimal, thermodynamically plausible set of microreversible chemical equations that can be analyzed with respect to their energy requirements. However, to explore how such chemical systems might be engineered de novo, we also propose an extended version based on enzyme-driven compartmentalized reactions. Finally, we show how a purely DNA system, built upon the paradigm of DNA strand displacement, can realize neuronal dynamics. Our analysis provides a compelling blueprint for exploring autonomous learning in biological settings, bringing us closer to realizing real synthetic biological intelligence.

## Introduction

While intelligent behaviors are usually associated with higher organisms that have a nervous system, adaptive and protointelligent behaviors are well documented in unicellular organisms. Examples include sensing,^1−3^ chemotaxis,^4,5^ or diauxic growth.^6−8^ This begs the question whether it is possible to rationally build molecular systems that show protointelligent behaviors and can be used as machines to monitor or control their chemical environment at a microscopic scale. Systems of this type could find applications in areas such as drug delivery, bioprocessing, or biofabrication.

As a step in this direction, we will probe how artificial intelligence can be realized in molecular systems. More specifically, we will show how to realize artificial neurons, as they are widely used in computer science as components of neural networks.^9^ Individual artificial neurons are simple machines but nevertheless show a remarkable ability to learn from observation. For the purpose of this article, we will consider a particular type of neuron, a spiking neuron (SN). SNs are widely used in machine learning,^10,11^ and it is well known that they have significant learning capabilities^12,13^ including principal component analysis,^14^ recognition of handwriting,^15^ or classification of fighter planes.^16^ There are a number of different models of SNs in the literature. Commonly a SN has an internal state, usually represented by a positive real number. The internal state may decay, which means that it reduces over time with some rate. The internal state variable increases when the SN receives a stimulus (an input spike) via one of its *N* input channels. Importantly, these input channels are weighted. The higher the weight, the more the internal state variable increases following an input spike through this channel. This weighting is crucial for the behaviors of the neuron. Consequently, “learning”, in the context of neural networks, normally means adjusting the weights.

There have been numerous attempts to build neurons in chemical systems. The earliest dates back to the 1980s by Okamoto and collaborators,^17^ who showed that certain biochemical systems implement the McCulloch–Pitts neuronic equations. Later, a mathematical description of a neuron was proposed,^18^ but this system had no ability to learn. Banda et al.^19^ used artificial chemistry to emulate an artificial neuron and a fully fledged feed-forward neural network^20^ which could solve the XOR problem. Their model requires regular interventions by outside operators, however. Besides these simulation studies, there have also been attempts to implement learning in vivo,^21−24^ but again, these systems are not autonomous: they rely on iterative measurement and manipulation protocols, which limit their practical deployment as computing machines within a molecular environment.

An attractive concept of learning that avoids the need to monitor the molecular neurons is Hebbian learning. This concept originated from neuroscience but is now widely used in artificial intelligence to train neural networks. The basic idea of Hebbian learning is that the connection between neurons that fire at the same time is strengthened. This update scheme is attractive because unlike many other learning algorithms, it does not require evaluating an objective function, which would be difficult to achieve in general with chemical networks.

To illustrate the basic idea of Hebbian learning—or associative learning as it is often called when there are only two input channels—consider a neuron with two inputs *A*_1_ and *A*_2_. Let the weights associated with the inputs be set such that (an output firing of) the neuron is triggered whenever *A*_1_ fires but not when *A*_2_ fires. Assume now that *A*_2_ fires usually at around the same time as *A*_1_. Then, its weights will be strengthened by the Hebbian rule because of the coincidence of *A*_1_ and *A*_2_. Eventually, the weights of the second channel will have increased sufficiently such that firing of *A*_2_ on its own will be sufficient to trigger an output.

Molecular models of Hebbian learning have been proposed before. A biochemical model of associative learning was proposed by Fernando and co-workers.^25^ Their model is fully autonomous, but it is also inflexible. Association is learned after just a single coincidence, and hence, the model is unable to detect statistical correlations robustly. Moreover, the system cannot forget the association between the inputs. McGregor et al.^26^ introduced an improved design with systems that were found by evolutionary processes. A biochemically more plausible system was proposed by Solé and co-workers,^27^ but this system is also limited to learning two coinciding inputs and relies on an explicit operator manipulation in order to forget past associations.

In this article we will propose a fully autonomous chemical artificial neuron, henceforth referred to as CN, Table 1, that goes beyond the state of the art in that it can learn statistical relations between an arbitrary number of inputs. The CN is also able to forget learned associations and as such can adapt to new observations without any intervention by an external observer. Via each of its input channels the CN can accept boli, which is the injection of a certain amount of chemical species, representing the input spikes of simulated neurons. The CN will “learn” the statistical biases of the input boli in the sense that the abundance of some of its constituent species, which play an analogous role to neuronal weights, reflect statistical biases of the boli. In particular, we consider two types of biases. (i) Frequency biases (FB): one or more input channels of the CN receive boli at different rates. (ii) Time correlations (TC): two or more input channels are correlated in time. The TC task can be understood as a direct generalization of associative learning with an arbitrary number of input channels.

**Table 1:** List of Acronyms

acronym	definition
CN	chemical neuron
c-CN	compartmentalized chemical neuron
d-CN	DNA chemical neuron
FB	frequency biases
TC	time correlations
DSD	DNA strand displacement

We will propose three different versions of the CN. The first (basic) version will be the CN itself, which is a minimal set of chemical reactions. It is also thermodynamically consistent in that it comprises only microreversible reactions with mass-action kinetics. This first version, while compact, assumes a high degree of enzymatic multiplicity which is unlikely to be realizable. Therefore, we shall propose a second version of the model which is not thermodynamically explicit but biologically plausible in the sense that it can be formulated in terms of known biochemical motifs. The main difference between this and the previous system is that the former is compartmentalized. Henceforth, this compartmentalized system will be referred to as c-CN.

We also propose d-CN, a version of the CN that is formulated using DNA strand displacement (DSD),^28^ a type of DNA-based computing. DSD is a molecular computing paradigm based entirely on interactions of DNA strands and Watson–Crick complementarity and is biocompatible. By this we mean that DSD computers can, in principle, be injected into organisms and interact with their biochemistry^29^ and therefore have potential to be used to control molecular systems. It has been shown that DSD systems are capable of universal computation^30^ and indeed that any chemical reaction network can be emulated in DSD.^31,32^ From a practical point of view, it is relatively easy to experimentally realize DSD systems, and their behavior can also be accurately predicted^33,34,37^ using simulation software such as Visual DSD^28^ or Peppercorn.^35^ There is now also a wealth of computational methods and tools for designing DNA-based circuits.^36,37^

Given these properties, there have been a number of attempts to build intelligent DSD systems. Examples include linear-threshold circuits, logic gates,^30,38^ switches,^39^ oscillators,^40^ and consensus algorithms.^32^

There were also some attempts to emulate neural networks in DSD: Qian et al.^41^ proposed a Hopfield network which has the ability to complete partially shown patterns. However, because the weights connecting individual neurons were hard coded into the system, the system was unable to learn. Networks of perceptron-like neurons with competitive winner-take-all architectures have also been proposed^42,43^ and show how to use DSD reaction networks to classify patterns, such as MNIST handwritten digits.^44^ However, learning is external to these systems; weights have to be determined before building the DNA circuit and are then hard coded into the design.

Supervised learning in DSD was proposed by Lakin and collaborators.^45^ They used a two-concentration multiplier circuit motif in order to model the gradient descent weight update rule. However, this approach requires an external observer to provide constant feedback. From the perspective of implementing artificial protointelligence in biochemistry, none of the above approaches can be used as a fully autonomous component of a molecular learning system in the sense that they can operate independently of constant external maintenance.

## Results

In the first part of this section, we describe the microreversible chemical reactions that constitute the CN. Next, we demonstrate that the system of reactions behaves like a spiking neuron, and we analyze the key parameters that determine the performance of the system. In the subsequent section, we describe c-CN, which lends itself more easily to experimental implementation. Finally, we discuss how DNA strand displacement can be used to construct the d-CN.

### Chemical Neuron—Minimal Model

#### Overview

**Figure 1:**
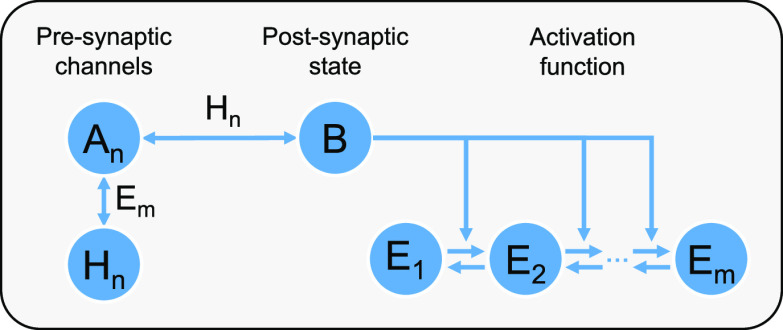
Graphical representation of the minimal model of the CN.

We model the CN as a set of microreversible elementary chemical reactions obeying mass-action kinetics (Table 2, Figure 1). Microreversibility makes the model thermodynamically consistent. The system is best understood by thinking of each molecular species *A*_*i*_ as an input to the system via channel *i*. The inputs are provided in a form of boli, which is defined as a fixed amount of molecules introduced to the system at the time of the input. The weight equivalent of the *i*th input channel of the CN is the abundance of the species *H*_*i*_. The species  is the activated form of *E* and plays a dual role. It is (i) the learning signal, which indicates that a weight update should take place, and (ii) the output of the CN, which could be coupled to further neurons downstream. The internal state of the CN, which acts as a memory for the system, is represented by the abundance of the molecular species *B*. We now proceed by discussing each reaction in Table 2 in turn.

**Table 2:** List of Chemical Reactions Constituting the CN

function	reaction(s)
input	
	
activation function	
	
learning	
	
leak	
	

#### Input

We assume here that the CN has *N* different species of input molecules *A*_1_, ..., *A*_*N*_. These represent the *N* input channels, each of which is associated with a corresponding weight *H*_1_, ...,*H*_*N*_. The weight molecules are the interpretable output of the neuron in the sense that the abundance of the *H*_*i*_ molecules will reflect statistical biases in the input. The input is always provided as an exponentially decaying bolus at a particular time *t*_*i*_^*s*^, where *s* is a label for individual spikes. Concretely, this means that at time *t* = *t*_*i*_^*s*^ the CN is brought into contact with a reservoir consisting of β (unmodeled) precursor molecules *I*_*i*_ that then decay into *A*_*i*_ molecules with a rate constant κ > 0. A particular consequence of this is that the *A*_*i*_ are not added instantaneously but will enter the system over a certain time. This particular procedure is a model choice that has been made for convenience. Different choices are possible and would not impact on the results to be presented. The important point is that the input signal to channel *i* is a bolus of quantity *A*_*i*_ and occurs at a particular time *t*. This enables the system to reach a steady state provided that the input is stationary.

The basic idea of the CN is that input boli *A*_*i*_ are converted into internal state molecules *B*. This reaction takes a catalyzed as well as an uncatalyzed form. The uncatalyzed reaction  is necessary in order to allow the system to learn to react in response to new stimulus, even when the weight associated with a given channel decayed to 0. In the case of the catalyzed reaction, the channel-specific *H*_*i*_ molecules play the role of the catalyst. Thus, the speed of conversion depends on the amount of weight *H*_*i*_. If at any one time there is enough of *B* in the system then the learning signal  is created by activating *E* molecules. Once the learning signal is present, some of the *A*_*i*_ are converted into weight molecules, such that the weight of the particular input channel increases. This realizes Hebbian learning in the sense that the coincidence of inputs *A*_*i*_ and output  activates weight increases following the well-known Hebbian tenet “What fires together, wires together”.

#### Activation Function

The link between the internal state molecules *B* and the learning signal is often called the activation function. In spiking neurons, as they are used in artificial intelligence, this activation is usually a threshold function. The neuron triggers an output if the internal state crosses a threshold value. In chemical realizations, such a threshold function is difficult to realize. Throughout this contribution, our systems are parametrized such that the dynamics of the system is dominated by noise. Molecular abundances are therefore noisy. As a consequence, the activation function has to be seen as the probability to observe the activated form  as a function of the abundance of *B*.

An ideal activation function would be a step function, but physical realization will necessarily need to approximate the step function by a continuous function, for example, a sigmoid. In the CN, this is realized as follows. Each of the *E* molecules has *m* binding sites for the internal state molecules *B*. Once all *m* binding sites are occupied, *E* is converted into its active form . We make the simplifying assumption that the conversion from *E* to  is instantaneous once the last *B* binds. Similarly, if a *B* molecule unbinds then the  changes immediately to *E*. In this model, the balance between  and *E* molecules depends on the binding and unbinding rates of *B*. We assume that there is a cooperative interaction between the *B* molecules such that unbinding of *B* from  is much slower than unbinding from *E*. With an appropriate choice of rate constants, this system is known to display ultrasensitivity, i.e., the probability for the fully occupied form of the ligand chain () to exist transitions rapidly from close to 0 to close to 1 as the concentration of ligands approaches a threshold value ϑ ≈ *k*_+_/*k*_–_. The dynamics of such systems is often approximated by the so-called Hill kinetics. It can be shown that the maximal Hill exponent that can be achieved by such a system is *m*.^46^ This means that the chain-length *m*, which we henceforth shall refer to as the “nonlinearity”, controls the steepness of the activation function of . In the limiting case of *m* = ∞, this will be a step function, whereby the probability to observe  is 0 if the abundance of *B* is below a threshold and 1 otherwise. We are limited here to finite values of *m* > 0. In this case, the function is sigmoidal or a saturating function in the case of *m* = 1. The parameter *m* and hence the steepness of the activation function will turn out to be crucial factors determining the computational properties of the CN.

#### Learning

**Figure 2:**
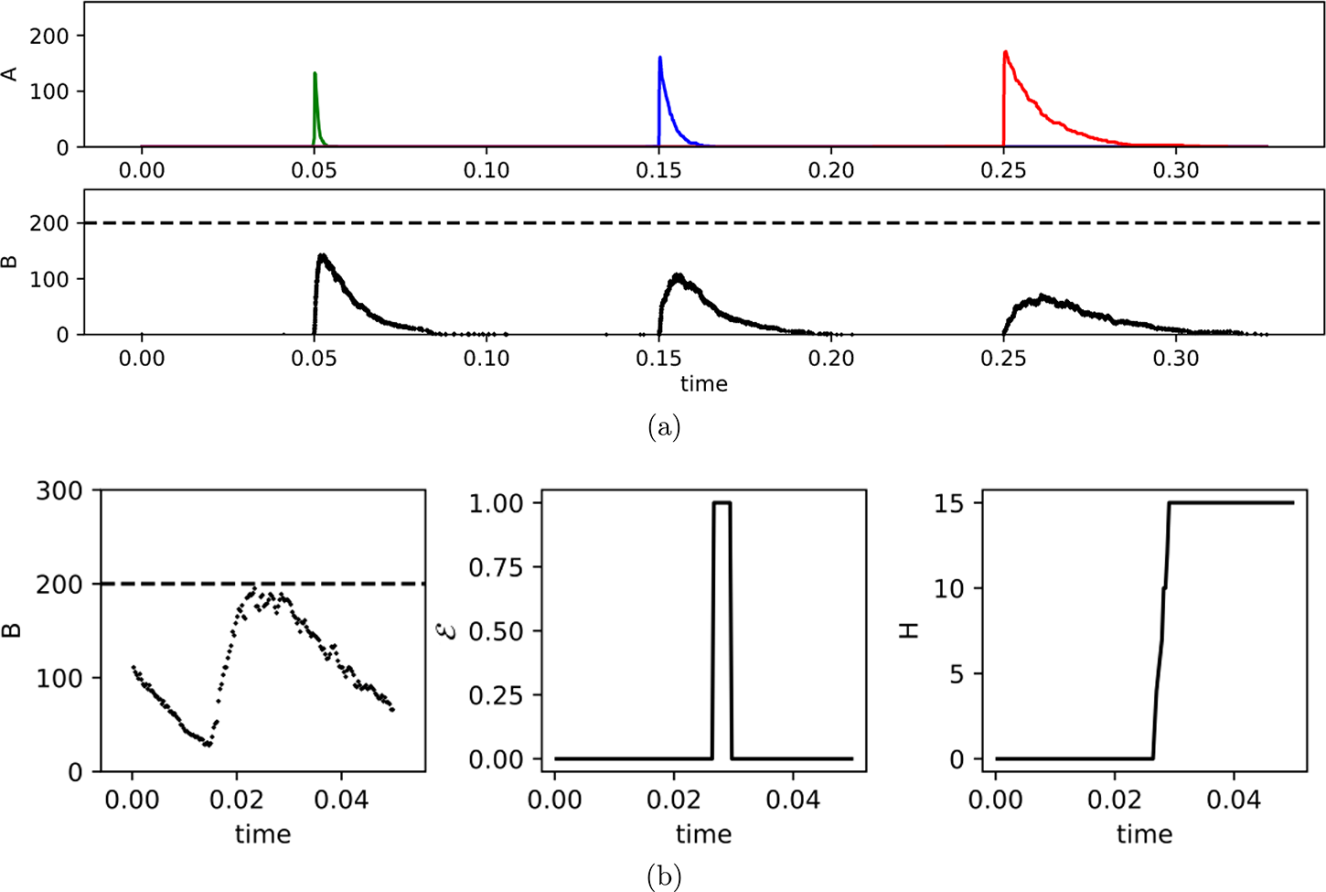
(a) Example of three inputs of uniform size received from 3 different channels. Each input shown in the second graph has a different weight associated it: *H*_green_ = 250, *H*_blue_ = 50, and *H*_red_ = 0. *H* molecules act as a catalyst in the  reaction, hence the change in the function of *B* molecules over time for each of the inputs. The higher the amount of *H*, the higher is the peak of *B* molecules caused by a particular input. Moreover, with the increase in weights, the function of inputs also changes. The higher the amount of *H*, the quicker its corresponding *A* dissipates. (b) Example simulation showing the core idea of the CN dynamics. Graphs show the internal state *B*, learning signal , and weight *H* for a single channel. We assume a bolus provided at time *t* = 0.015. This causes the internal state to go up and reach the threshold. Learning signal is triggered at around *t* = 0.03, and consequently, the weight is increased by (in this case) 15 molecules of *H*.

In neural networks, “learning” is usually associated with the update of weights. Accordingly, in the case of the CN, learning is the change of abundances *H*_*i*_. The abundance can only increase if two conditions are fulfilled: (i) the learning signal  is present and (ii) there are still input molecules *A*_*i*_ in the system. In short, learning can only happen if input and output coincide, which is precisely the idea of Hebbian learning. For an illustrative example of how Hebbian learning works in the CN, see Figure 2.

#### Leak

Finally, we assume that the weight molecules *H*_*i*_ and the internal state molecules *B* decay, albeit at different rates. This is so that the weight abundances can reach a steady state; in addition, it enables the CN to forget past inputs and to adapt when the statistics of the input changes. We will assume that the decay of *H*_*i*_ is slow compared to the typical rate of input boli.

Throughout this paper we will assume that the dynamics of *A*, *B,* and *E* are fast compared to the change in concentration of *H*. This is a crucial assumption to allow the weights to capture long-term statistics of inputs; in particular, the weights should not be influenced by high-frequency noise present in the system. Furthermore, we also assume that the lifetime of  is short. For details of the parameters used, see Table S1.

#### Associative Learning

**Figure 3:**
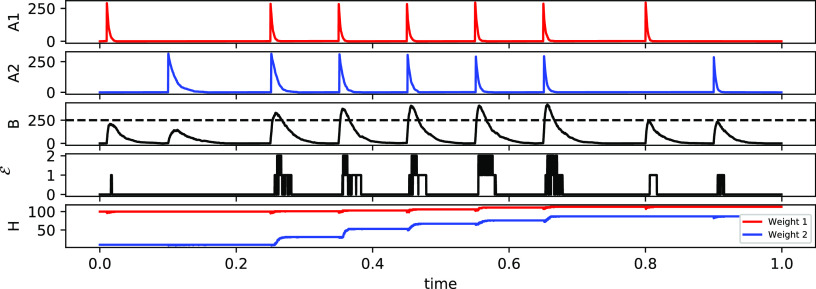
Associative learning in CN. First two graphs show inputs *A*_1_ and *A*_2_. Clearly, a single *A*_2_ does not lead to a sufficient increase of the internal state *B*, such that no learning signal is triggered. After a few coincidences of *A*_1_ and *A*_2_, weights *H*_2_ (last graph) have increased sufficiently for *A*_2_ to trigger a signal in its own at time *t* = 0.8. Note the increase in weights for the second channel after each coincidence.

We first demonstrate that the CN is capable of associative learning (Figure 3). To do this, we generate a CN with *N* = 2 input channels. Then, we initialize the CN with a high weight for the first channel (*H*_1_ = 100) and a low weight for the second channel (*H*_2_ = 0). Furthermore, we set the parameters of the model such that a bolus of *A*_1_ is sufficient to trigger an output but a bolus of *A*_2_, corresponding to stimulating the second channel, is not. This also means that presenting simultaneously both *A*_1_ and *A*_2_ triggers a learning signal and increases *H*_1_ and *H*_2_. If *A*_1_ and *A*_2_ coincide a few times then the weights of *A*_2_ have increased sufficiently so that a bolus of *A*_2_ can push the internal state of the system over the threshold on its own. This demonstrates associative learning. Note that unlike some previous molecular models of associative learning (e.g., ref (25)), the CN requires several coincidences before it learns the association. It is thus robust against noise.

This means that the CN can also readily unlearn the correlation if input patterns change (see Figures S4 and S5). There are two mechanisms in the system that ensure that the neuron is able to continuously learn new input statistics. These are (i) the decay of the weights, which ensures a rate of forgetting, and (ii) the uncatalyzed reaction *A*_*n*_ to *B*, which allows the system to learn to react in response to new stimulus, even when the weight associated with a given channel decayed to 0.

#### Full Hebbian Learning

We now show that the ability of the CN to learn extends to full Hebbian learning with an arbitrary number of *N* input channels. First, we consider the FB task, where the CN should detect input channels that fire at a higher frequency than others. To do this, we provide random boli to each of the *N* input channels. Random here means that the waiting time between two successive boli of *A*_*i*_ is distributed according to an exponential distribution with parameter 1/*f*_*i*_, where *f*_*i*_ is the frequency of the input boli to channel *i*. The CN should then detect the difference in frequencies *f*_*i*_ between input channels. We consider the FB task as solved if (after a transient period) the ordering of the abundances of weights reflects the input frequencies, i.e., the number of *H*_*i*_ should be higher than the number of *H*_*j*_ if *f*_*i*_ > *f*_*j*_. Below we will show, using a number of example simulations, that the CN is indeed able to show the desired behavior. Later, we will probe in more detail how the response of the system depends on its parametrization and the strength of the input signal.

**Figure 4:**
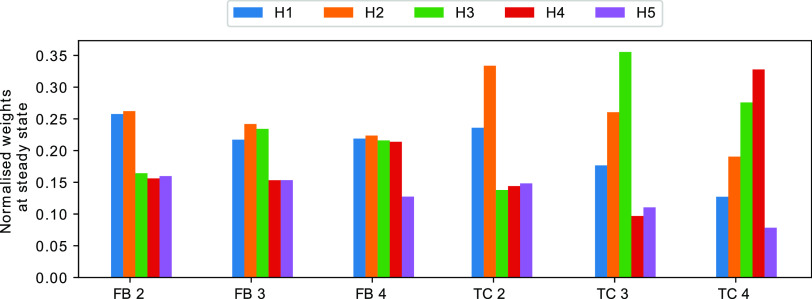
Normalized weights for a variety of TC and FB tasks. First (blue) bar refers to the first weight, second (orange) to the weight for the second channel, and so on. Each value represents the average over 300 time units of a single simulation. Data was only collected after the weights reached the steady state (after 700 time units). In all experiments, we set the number of *E*_1_ molecules at the start of the simulation to 40. Nonlinearity was set to *m* = 5 for the TC and *m* = 1 for FB.

In order to test a CN with multiple inputs (*N* = 5, *m* = 1), we consider 3 variants of the FB task. First, we assume that boli to the first two input channels come at a frequency of 4 Hz, whereas channels 3, 4, and 5 fire at a frequency of 2 Hz; we call this variant FB 2. Similarly, for FB 3 and FB 4, the first 3 and 4 channels, respectively, fire at the higher frequency. Figure 4 shows the steady state weights for each of the three tasks. As expected, in each of the experiments, the weights of the high-frequency inputs are higher when compared to the low-frequency inputs. We conclude that the CN can work as a frequency detector at least for some parametrizations.

The other scenario that we will investigate is the TC task, which is the direct generalization of the associative learning task to an arbitrary number of input channels. For this problem we assume that all input frequencies are the same, i.e., *f*_*i*_ = *f*_*j*_ for all *i*,*j* ≤ *N*. Instead of differences in frequency, we allow temporal correlations between input boli of some channels. If *A*_1_ and *A*_2_ are temporally correlated then each bolus of *A*_1_ is followed by a bolus of *A*_2_ after a time period of δ + ξ, with δ being a fixed number and ξ a random variable drawn from a normal distribution with μ = 0 and σ^2^ = 0.0001 for each bolus. In all simulations, the input frequency of all channels is set to 2 Hz.

The CN can solve the TC task in the sense that, after a transient period, the weights indicate which channels are correlated. They also indicate the temporal order implied by the correlation, i.e., if *A*_*i*_ tends to precede *A*_*j*_ then the abundance of weight *H*_*i*_ should be lower than the abundance of *H*_*j*_. Furthermore, if *A*_*i*_ is correlated with some other channel *k* but *A*_*j*_ is not then the abundance of *H*_*i*_ must be greater than that of *H*_*j*_.

In order to test whether the system is indeed able to detect TC biases, we again simulated a CN with *N* = 5 input channels and all weight molecules initialized to *H*_*i*_ = 0. We then determined the steady state weights in four different scenarios: there are correlations between (i) A_1_ and *A*_2_ (TC 2), (ii) A_1_, *A*_2_, and *A*_3_ (TC 3), and (iii) A_1_, *A*_2_, *A*_3_, and *A*_4_ (TC 4). The temporal order is always in ascending order of the index, such that in the last example, *A*_1_ occurs before *A*_2_, which in turn occurs before *A*_3_. We find that the behavior of the CN is as expected (Figure 4). At steady state the weights reflect the correlation between input channels, including the temporal ordering, thus allowing us to conclude that, at least for some parametrizations, the CN successfully identify temporal correlations.

#### Analysis of Activation Function Nonlinearity

The ability of the CN to perform in the TC task depends on its ability to detect coincidences. In this section, we will now analyze in more detail how this coincidence detection depends on the nonlinearity of the activation function, i.e., the parameter *m*. To do this, we consider two extreme cases: First, the case of minimal nonlinearity (i.e., *m* = 1), and second, the limiting (and hypothetical) case of maximal nonlinearity (i.e., *m* = ∞). This latter case would correspond to an activation function that is a step function. While a chemical neuron cannot realize a pure step function, considering the limiting case provides valuable insight.

We consider first this latter scenario with a CN with two inputs *A*_1_ and *A*_2_. In this case, there will be a learning signal  in the CN if the abundance of *B* crosses the threshold ϑ. Let us now assume that the parameters are set such that a single bolus of either *A*_1_ or *A*_2_ is not sufficient to push the abundance of *B* over the threshold but a coincidence of both is. In this scenario then we have the following.

- A single bolus of *A*_1_ will not lead to a threshold crossing. No learning signal is generated, and weights are not increased.
- If a bolus of *A*_1_ coincides with a bolus of *A*_2_ then this may lead to a crossing of the threshold of the internal state. A learning signal is generated. Weights for both input channels 1 and 2 are increased (although typically not by equal amounts).

Next, consider an activation function tuned to the opposite extreme, i.e., *m* = 1. It will still be true that both *A*_1_ and *A*_2_ are required to push the abundance of *B* across the threshold. However, the learning behavior of the CN will be different.

- A single bolus of *A*_1_ will not lead to a threshold crossing. A learning signal may still be generated even below the threshold because the activation function is not a strict step function. The weight *H*_1_ will increase by some amount, depending on the bolus size.
- If a bolus of *A*_1_ coincides with a bolus of *A*_2_ then this will lead to more learning signal being generated than in the case of *A*_1_ only. As a result, the weights for both input channels 1 and 2 are increased by more than if they had occurred separately.

These two extreme cases illustrate how the CN integrates over input. In the case of low nonlinearity, the weights of a channel will be a weighted sum over all input events of this channel. The weights will be higher for channels whose boli coincide often. On the other hand, a step-like activation function will integrate only over those events where the threshold was crossed, thus specifically detect coincidences. From this we can derive two conjectures.

- The higher the nonlinearity, the better the CN at detecting coincidences. Low nonlinearity still allows coincidence detection but in a much weaker form.
- As the bolus size increases, the CN will lose its ability to detect coincidences, especially when the bolus size is so large that a single bolus is sufficient to push the abundance of *B* over the threshold. In this case, a single input spike can saturate the activation function, thus undermining the ability of the system to detect coincidences effectively.

**Figure 5:**
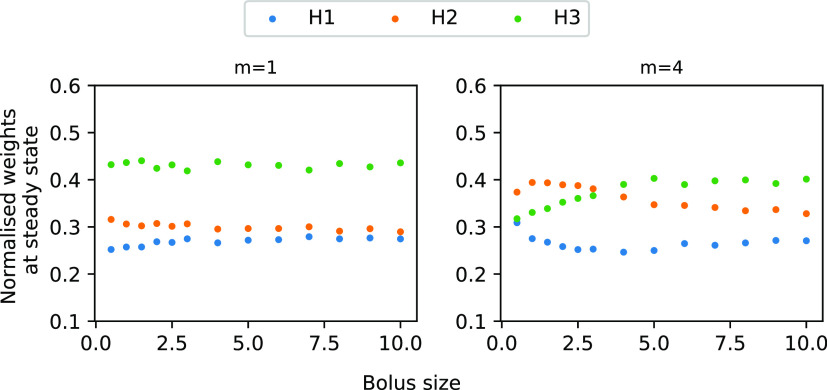
Steady state weights as a function of bolus size for a CN with 3 inputs. Input *A*_3_ (green) is provided at 4 Hz, *A*_1_ and *A*_2_ are correlated with δ = 0.0047, but they are only provided at 2 Hz. Graph shows the normalized weights at steady state corresponding to the input channels for different bolus sizes (here reported as a fraction of the threshold). From left to right, bolus size increases. For *m* = 1, the system detects the higher frequency of *A*_1_ as indicated by its high weight. It also differentiates between the correlated inputs but with weaker signal. As the bolus size increases, the neuron maintains its ability to recognize FB but can no longer detect TC, i.e., *H*_1_ and *H*_2_ have the same abundance. For the higher nonlinearity (*m* = 4), the system detects the TC (*H*_2_ has a higher abundance than *H*_1_). As the bolus size increases, it detects the FB but its ability to detect TC decreases.

In order to check these conjectures, we simulated a version of the CN with 3 inputs, where *A*_1_ and *A*_2_ are correlated and *A*_3_ fires at twice the frequency of *A*_1_ and *A*_2_. We considered the minimally nonlinear case (*m* = 1) and a moderate nonlinearity (*m* = 4), which shows the weights as a function of the bolus size (Figure 5). The minimal nonlinear CN detects both coincidences and frequency differences but loses its ability to detect coincidences as the bolus size increases. This is consistent with the above formulated hypothesis. In contrast, for the nonlinear CN and moderately low bolus sizes, the weights indicate the coincidences strongly (i.e., the weights *H*_2_ are highest) and less so the FB. As the bolus size increases, the nonlinear CN loses its ability to detect coincidences and becomes a frequency detector, as conjectured.

**Figure 6:**
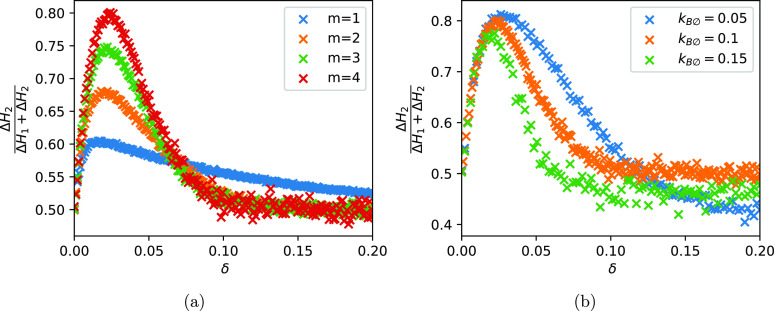
Differential weight increase for different nonlinearities. For both graphs, the points were computed as follows: We simulated a CN with two input channels only. We set the initial condition to *H*_1_, *H*_2_ = 0. At time *t* = 0, we provided a bolus of *A*_1_, and after a time period of δ, we provided the bolus *A*_2_. We then continued the simulation for another 0.2 time units. *y* axis records the relative increase of *H*_2_ over 0.2 time units averaged over 1000 repetitions. (a) We stimulate channel 1 followed by channel 2 after a time period of δ. Then, we measure the amount by which weights *H*_1_ and *H*_2_ were increased and record the fraction. Value of 1 means that only the second input channel received weight accumulation. Value of 0.5 means that the weights of both channels were updated equally. (b) Same but for different removal rates of *B*. The faster the removal, the more specific the coincidence detection, i.e., inputs need to occur within a narrower window.

Next, we check how the coincidence detection depends on the time delay between the correlated signals. To do this, we created a scenario where we provided two boli to the system. The first bolus *A*_1_ comes at a fixed time and the second one a fixed time period δ thereafter. We then vary the length of δ and record the accumulation of weights *H*_2_ as a fraction of the total weight accumulation. Figure 6 shows the average weight accumulation per spike event. It confirms that the CN with low nonlinearity is less sensitive to short coincidences than the CN with higher *m*. However, it can detect coincidences over a wider range of lag durations. This means that for higher nonlinearities, the differential weight update becomes more specific but also more limited in its ability to detect coincidences that are far apart. In the particular case of δ > 0.1, the CN with *m* > 1 does not detect any coincidences any more whereas the case of *m* = 1 shows some differential weight update throughout.

Next, we tested the conjecture that the TC can be solved more effectively by the CN when the nonlinearity is higher. To do this, we generated a CN with *N* = 5 input channels on the TC 2 task. We then trained the CN for nonlinearities *m* = 1, ..., 10. As a measure of the ability of the system to distinguish the weights, we used the index of dispersion, i.e., the standard deviation divided by the mean of the weights. A higher index of dispersion indicates more heterogeneity of the weights and hence a better ability of the system to discriminate between the biased and the unbiased input channels.

**Figure 7:**
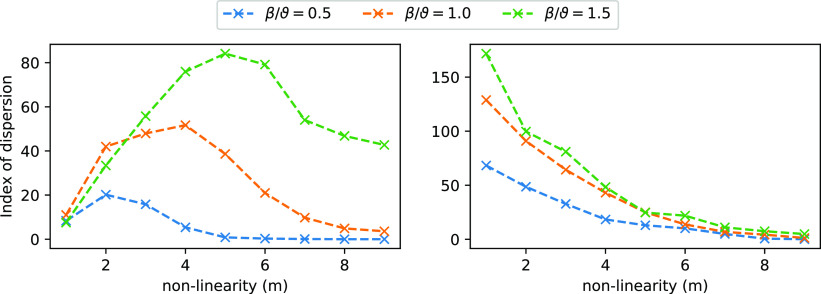
Index of dispersion for different bolus sizes β expressed as a fraction of the threshold ϑ. We show TC 2 (left) and FB 2 (right). Index of dispersion measures how different the steady state weights are from one another and hence indicates how well the CN distinguished between input channels. Completely unbiased input would give an index of dispersion of ∼0. Graph shows that for the TC task, there is an optimal nonlinearity. Increasing the bolus size increases the optimal nonlinearity, which is consistent with the fact that the optimum is due to resource starvation.

Consistent with our hypothesis, we found that the ability to distinguish temporarily correlated inputs increases with the nonlinearity. However, it does so only up to a point (the optimal nonlinearity), beyond which the index of dispersion reduces again (Figure 7). Increasing the bolus size, i.e., increasing the number of *A*_*i*_ that are contained within a single bolus, shifts the optimal nonlinearity to the right. This suggests that the decline in the performance of the CN for higher chain lengths is due to a resource starvation. The realization of the sigmoidal function, i.e., the thresholding reactions in Table 2, withdraws *m* molecules of *B* from the system. As a consequence, the CN is no longer able to represent its internal state efficiently and the activation function is distorted. If the total abundance of *B* is high compared to *E* then this effect is negligible. We conclude that there is a resource cost associated with computing nonlinearity. The higher *m*, the higher the bolus size required to faithfully realize the activation function. As an aside, we note that other designs for the system are also possible. For example, *B* molecules could be used catalytically. Nevertheless, such systems would also face different trade-offs. The system presented here was one of many designs that we tested and provided the most desirable properties for learning temporal patterns.

While the TC task requires nonlinearity, the FB task does not. This can be understood acknowledging that the FB task is fundamentally about integrating over input, which can be done naturally in chemical systems. Indeed, it can be done by systems that are much simpler than the CN. For example, the minimal system to detect FB bias is . For appropriately chosen values of *d*, the steady state value of *A*_*i*_ would then reflect the input frequency. To understand this, note that the input frequency determines the rate of increase of *A*_*i*_. This rate divided by the decay rate constant *d* then determines the steady state abundance of *A*_*i*_, such that *A*_*i*_ trivially records its own frequency. This system is the minimal and ideal frequency detector.

The CN itself is not an ideal frequency detector because all weight updates are mediated by the internal state *B*. Hence, the weights are always convolutions over all inputs. The weights thus reflect both frequency bias and temporal correlations. In many applications this may be desired, but sometimes it may not be. We now consider the conditions necessary to turn the CN into a pure frequency detector, i.e., a system that indicates only FB but not TC. One possibility is to set the parameters such that the CN approximates the minimal system. This could be achieved by setting *k*_BA_ ≪ *k*_AB_ and all other rate constants very high in comparison to *k*_AB_. The second possibility is to tune the CN such that a single bolus saturates the threshold. In this case, the strength of the learning signal does not depend on the number of boli that are active at any one time. A single bolus will trigger the maximal learning signal. This is confirmed by Figure 5, which shows that as the bolus size increases, the system becomes increasingly unable to detect temporal correlations but remains sensitive to frequency differences.

### c-CN: CN with Compartments

The CN, as presented in Table 2, is thermodynamically plausible and has the benefit of being easy to simulate and analyze. However, it is biologically implausible. As written in Table 2, the molecular species *A*_*i*_, *H*_*i*_, and *B* would have to be interpreted as conformations of the same molecule with different energy levels. In addition, we require that these different conformations have specific enzymatic properties. Molecules with the required properties are not known currently, and it is unlikely that they will be discovered or engineered in the near future.

As we will show now, it is possible to reinterpret the reaction network that constitutes the CN (Table 2) so as to get a model whose elements are easily recognizable as common biochemical motifs. This requires only relatively minor adjustments of the reactions themselves but a fundamental reinterpretation of what the reactions mean.

**Figure 8:**
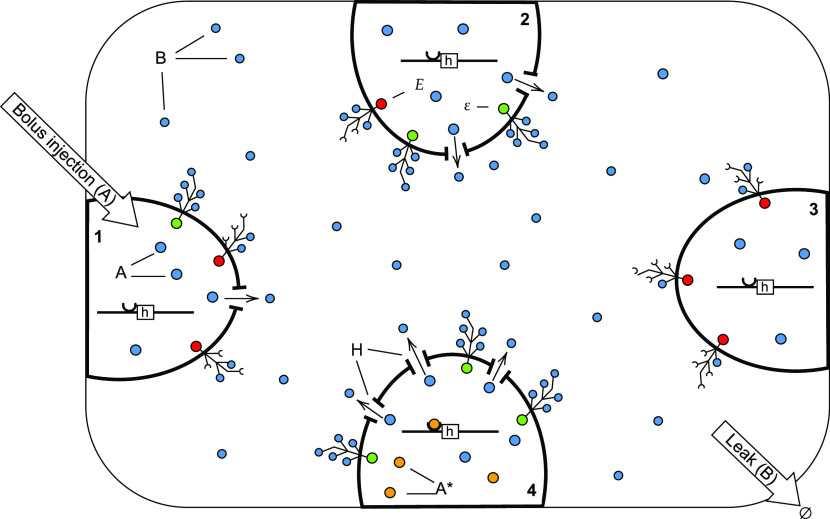
Graphical representation of a c-CN. *A*_*i*_ and *A*_*j*_ are the same molecular species but contained in different compartments *i* and *j*, respectively. We allow for an activated form of *A*, denoted by A*, which binds to the promotor site of *h* and activates its expression. *H* is an active transporter molecule for *A*. Once exported to the extracellular space, an *A*_*i*_ molecules become a molecule of *B*. We assume that each compartment has a transmembrane protein *E* with *m* extracellular binding sites. If all *m* binding sites are occupied by *B* then the internal site becomes active (indicated by green) and can catalyze the activation of *A*.

The main difference we introduce is that the new model is compartmentalized (Figure 8). While in the basic model the indices of *A*_*i*_ and *H*_*i*_ referred to different species that exist in the same volume, it should now be interpreted as the same species but living in different compartments. This means that *A*_*i*_ and *A*_*j*_ are the same type of molecule but located in compartments *i* and *j*, respectively. Similarly, *H*_*i*_ and *H*_*j*_ are the same species. All compartments *i* and *j* are themselves enveloped in a further compartment (the “extracellular space”). The internal state species *B* is the same as *A*_*i*_ but located in the extracellular space. From here on, we will refer to this reinterpreted model as the c-CN. It is formally described by the reactions in Table 3.

**Table 3:** List of Chemical Reactions Constituting the c-CN^a^

function	reaction(s)
input	
	
activation function	
	
weight accumulation	
	
	
	
	
leak	
	

a Molecular species *A*, *E*, , *h*_0_, *h*, and *H* are compartmentalized. Each compartment has a gene *h*_0_ which when activated by A* can express a transporter *H*.

Input to channel *i* is provided by boli of the molecular species *A* into the compartment *i*. A novelty of c-CN when compared to CN is that it has an activated form of *A*, denoted by A*. The conversion from *A* to A* is catalyzed by the learning signal . Also new is that each compartment contains a gene *h* that codes for the molecule *H* (we suppress the index indicating the compartment). Expression of the gene is activated by A* binding to the promoter site of *h*. We also allow a low leak expression by the inactivated gene (denoted as *h*_0_ in Table 3). Gene activation of this type is frequently modeled using Michaelis–Menten kinetics, thus reproducing in good approximation the corresponding enzyme kinetics in the CN. The molecules of type *H* are now transporters for *A*. We then interpret the conversion of *A*_*i*_ to *B* as export of *A* from compartment *i* to the extracellular space. The rate of export of *A* is specific to each compartment in that it depends on the abundance of *H* in this compartment. Finally, we interpret the *E* molecules as transmembrane proteins that are embedded in the membrane of each compartment. Their extracellular part has *m* binding sites for *B* molecules which bind cooperatively. When all sites are occupied, the intracellular part is activated, i.e., becomes . In its activated form it can convert *A* to A*.

Another difference between the two versions of the models is that the molecule *E* is now specific to each membrane. The minimum number of copies of *E* is thus *N*, whereas in the basic model a single copy of *E* at time *t* = 0 could be sufficient. This has two consequences. First, at any particular time the number of occupied binding sites will typically be different across the different *N* compartments. This is a source of additional variability. Moreover, since the number of copies of *E* is higher than that in CN, the c-CN is more susceptible to starvation of *B* as a result of the extracellular binding sites withdrawing molecules from the outer compartment. Both of these potential problems can be overcome by tuning the model such that the abundance of *B* molecules is high in comparison to *E* molecules.

**Figure 9:**
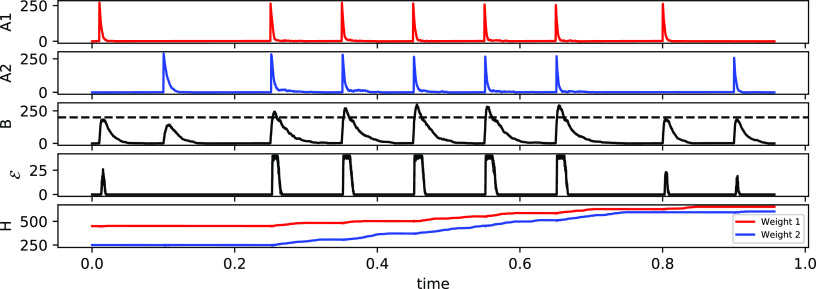
Same as Figure 3 but for c-CN. For the parameters used, see Table S2. For this experiment, we approximated the ligand kinetics by a Hill function in order to speed up the simulations.

**Figure 10:**
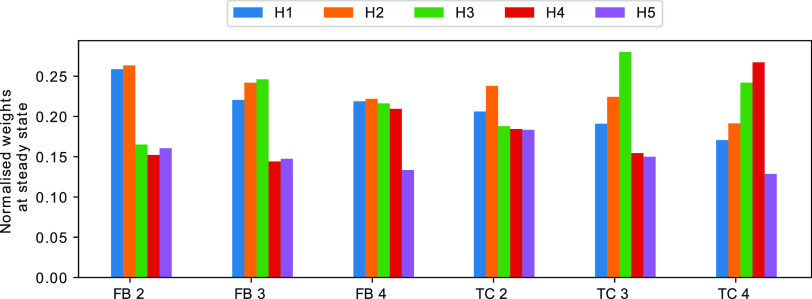
Same as Figure 4 but for c-CN. Experiments approximated the ligand dynamics by a Hill function in order to speed up the simulations.

This highlights that the differences between the basic CN and c-CN are deeper than the list of reaction suggests. Our simulations, however, confirm that the c-CN supports associative learning (Figure 9) and full Hebbian learning (Figure 10) just as the basic CN provided that the parameters are set appropriately.

### d-CN: Chemical Neuron in DNA

We now show how to emulate the chemical reaction network of Table 2 using DNA strand displacement (DSD).^28^ This is interesting because the experimental realization of DSD systems is straightforward and predictable when compared to biochemical reaction networks.

The basic idea of DNA-based computation is that double-stranded DNA molecules with an overhang on one strand—often called the toehold—can interact with single-stranded DNA that contains the Watson–Crick complement of the toehold via partial or total displacement of the existing complement. DNA-based systems are typically analyzed on two levels: the sequence level and domain level. The former involves the study of interactions between individual nucleotide pairs, while the latter focuses on the interactions between domains. Here, domains are sequences of nucleotides of varied length. There are two types of domains which are differentiated by their length. Short domains or toeholds are between 4 and 10 nucleotides and are assumed to be able to bind and unbind from complementary strands. Long domains, or recognition domains, are at least 20 nucleotides in length and assumed to bind irreversibly. DSD is a domain-level mechanism for performing computational tasks with DNA via two basic operations: toehold-mediated branch migration and strand displacement.

#### Implementing the d-CN Using Two-Domain DSD

In order to emulate the chemical neuron in DNA, we will focus here on two-domain strand displacement,^32,36^ where each molecular species comprises a toehold and a long domain only. These species can interact with double-stranded gates which facilitate the computation. Restricting computation to two-domain strands helps to protect against unexpected interactions between single-stranded species, which can occur with more complex molecules. Also, as all double-stranded structures are stable and can only change once a single-stranded component has bound, there is no possibility for gate complexes to polymerize and interact with each other.

Here, we will be using the standard syntax of the Visual DSD programming language^28^ to describe the species present in our system. We denote double-stranded molecules as [r], where its upper strand <r> is connected to a complementary lower strand {r*}. Each of the reactants and products in our system is an upper single-stranded molecule composed of a short toehold domain (annotated with a prefix t and an identifier ^) and a corresponding long domain <tr^ r>. We will refer to a short domain of a two-domain DSD strand *A*_*n*_ as ta and its corresponding long domain as an, where *n* is a channel index. Note that the toehold is not specific to the species index *n*, and therefore, the recognition of each input and weight strand is dependent on their long domains rather than their toeholds. We will use the same convention for all other channel-specific two-domain species. For a detailed description of the nucleotide structure and binding rates, see Tables S3 and S4 in the SI. The main two-domain strands that enable communication between different modules of the d-CN are shown in Table 5.

While there is a theoretical guarantee that any chemical reaction network can be mimicked by a DSD circuit,^32^ it is often difficult to find circuits. However, there are now a number of general design motifs with known behaviors in the literature. Here, we will make extensive use of the two-domain scheme, which introduces a Join–Fork motif to mimic a chemical reaction. While the abstract chemical system remains broadly similar to the CN model, there are some crucial differences (see Table 4). The general strategy we take to convert the CN to DSD is to translate each of the catalytic reactions in Table 4 into a Join–Fork gate.^32,36^ Subsequently, we will simulate the gates acting in concert.

**Table 4:** List of Reactions That Constitute the d-CN

function	reaction
signal integration	
weight accumulation	*A*_*n*_ + *E* ⇌ *E* + *H*_*n*_
signal modulation	
activation function	
	

**Table 5:** List of Key DNA Strands Which Facilitate Learning

name	signal	DSD species
input	*A*_*n*_	<ta^ an>
weights	*H*_*n*_	<th^ hn>
internal state	*B*	<tb^ b>
learning signal	*E*	<b tem^ b>
signal integration fuel	*Fsi*_*n*_	<tfsi^ fsin>

We first explain how we use the Join–Fork gates. For each reaction, a Join gate is able to bind the reactants and produces a translator strand. Then, the translator activates a Fork gate, which in turn releases the reaction products. Additional energy must be supplied to completely release all products from Fork gates, as the translator strand will only displace the first product. Appropriately designed helper strands are therefore placed in the solution to release subsequent products. After the first product has unbound, an exposed toehold is left, which can lead to unwanted side effects. To address this, we follow^32^ and extend the original design from ref (36) by incorporating an additional long domain on the left-hand side of the Fork gate, which upon binding an appropriate auxiliary molecule seals the gate to prevent rebinding of its outputs. Here, we extend all Join gates in an equivalent way to prevent rebinding of the translator strand. This addition allows us to avoid interactions of the double-stranded complexes with waste molecules.

In our design, binding of the translator immediately releases an *A*_*n*_ (<ta^ an>) strand, the first of the reaction products. The second product, *B* (<tb^ b>), is released upon binding of a Fork helper strand <b ta^>. Finally, the Fork_AB_ gate is sealed upon binding of the Fork seal strand <i tb^>. The pair of Join and Fork gates together consume 1 molecule for each of the reactants and produce 1 molecule for each of the products, ensuring equivalent stoichiometry to the abstract reaction.

**Figure 11:**
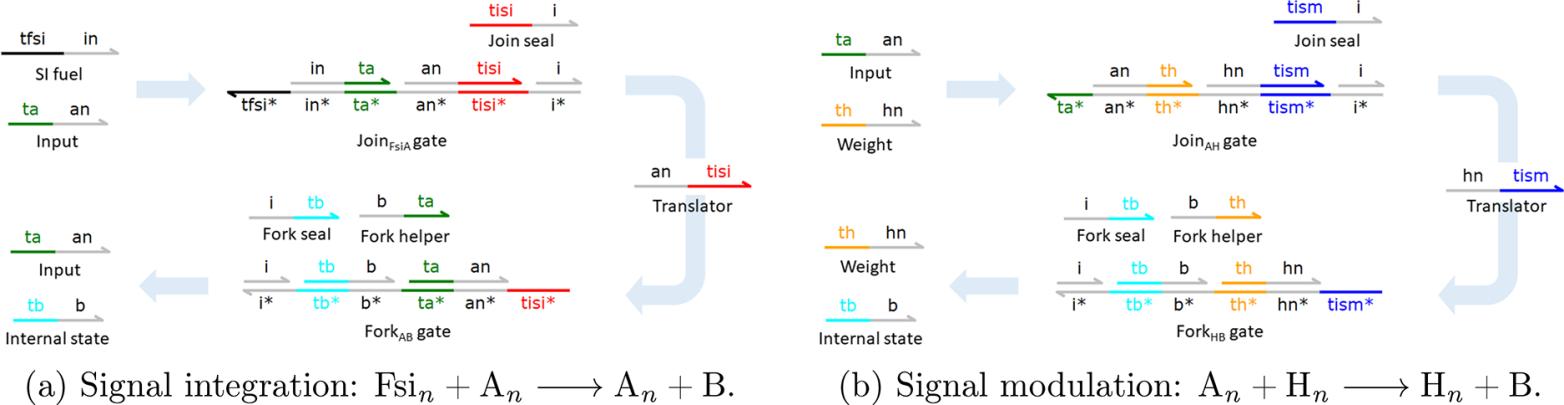
Mapping the CRN neuron to a DNA neuron. We use a two-domain Join–Fork gate to emulate each of the catalytic reactions in the CN (Table 4). In each case, a Join gate binds the two reactants in sequence, first displacing a waste molecule and second displacing a translator molecule, which triggers the corresponding Fork gate to release strands representing the reaction products. Translator displaces the first product, and then a Fork helper displaces the second product. Both Join and Fork gates can be sealed upon binding of an appropriate auxiliary strand (labeled Join seal and Fork seal), which displaces the final incumbent bound <i> strand.

In order to illustrate the mapping from the CN to DSD, we describe now in detail the reaction *Fsi*_*n*_ + *A*_*n*_ → *A*_*n*_ + *B* (Figure 11a), which serves as a representative of all 3 catalytic reactions in the d-CN. A Join*_AFsi_* gate is defined by a structure that enables the binding of *Fsi*_*n*_ and *A*_*n*_; the gate is only active if both input species are present. First, *Fsi*_*n*_ binds and displaces the incumbent bound <in ta^> molecule, exposing the ta^ toehold. This enables the binding of *A*_*n*_ (<ta^ an>), which then displaces the <an tisi^> translator strand, signaling that the reactants have been received and that the overall reaction can fire. The Join_*FsiA*_ gate is then sealed by the binding of <tisi^ i>, preventing rebinding of the translator and producing a further waste molecule <i>. The Fork_*AB*_ gate is designed in such a way that upon triggering by the translator strand of the corresponding Join gate it is able to release both product molecules.

#### Controlling the Activation Function Nonlinearity with Extended Polymers

The only reaction which takes a different form than a combination of Join and Fork gates is the activation function. We first describe the simplest case of an activation function with minimal nonlinearity, i.e., *m* = 1. In this case it takes the form {tb^*} [b te0^]: [b te1^]<b> or graphically: 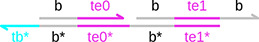. *B* molecules can bind to this compound; in doing so they expose the te0 short domain which allows for binding of *E*_0_. When *E*_0_ binds to the complex, it displaces a long domain b and releases the learning signal , which in the case of *m* = 1 is represented by three-domain species <b te1^ b>.

**Figure 12:**
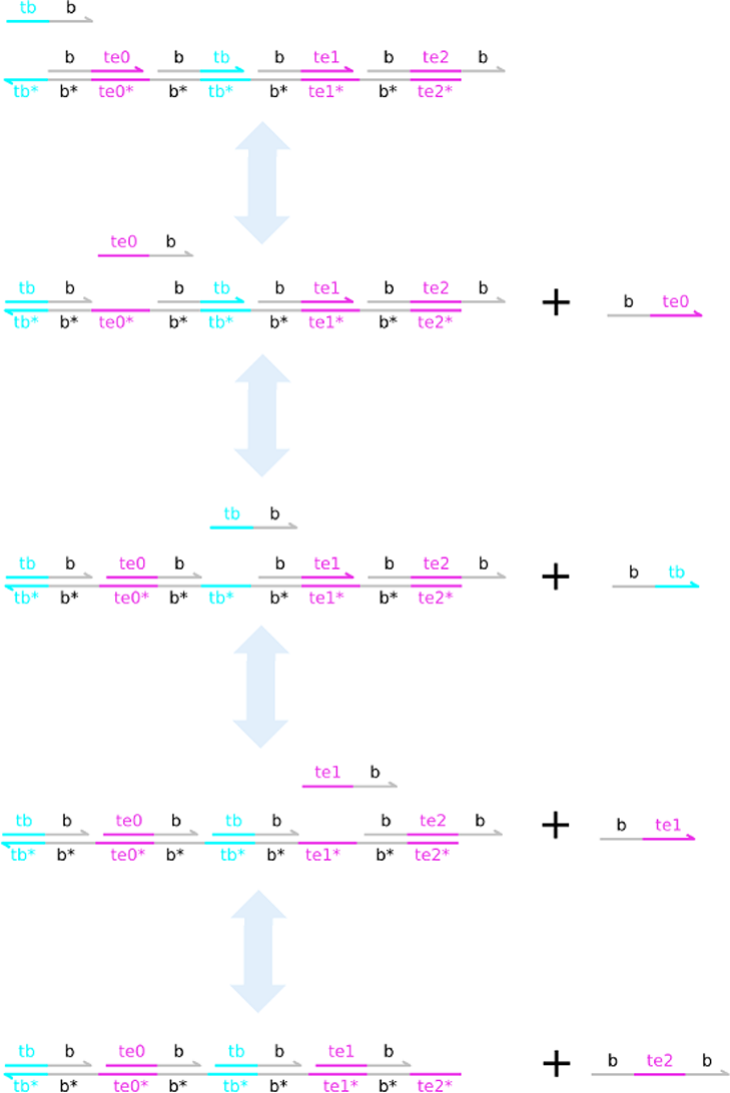
Activation function for *m* = 2 is modeled as a long polymer which accommodates for binding of *B* and subsequent *E* molecules to its surface. These two species can bind to the polymer in an alternate manner. First, the binding of *B* frees up a te0 toehold; next, the binding of *E*_1_ frees up a tb toehold, etc. Altogether, this process consumes *B* molecules. At the end of the process, a three-domain learning signal molecule  is produced. In the case of *m* = 2, this molecule takes the following form: <b te2^ b>. This mechanism can also run backward to produce *B* molecules.

This system can now be generalized to arbitrary integer values of *m* by extending the polymer with additional segments to accommodate for binding of more *B* and *E*_*m*_ molecules (Figure 12). We use segments of the form [b tb^]:[b tek^], where *k* is the index of the *k*th extra segment in the complex. Each new segment should be added before the last fragment which contains : [b te1^]<b>. In the case of *m* = 2, the activation function then is {tb^*} [b te0^]: [b tb^]: [b te1^]: [b te2^]<b> or graphically: 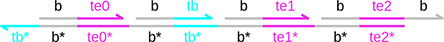.

**Figure 13:**
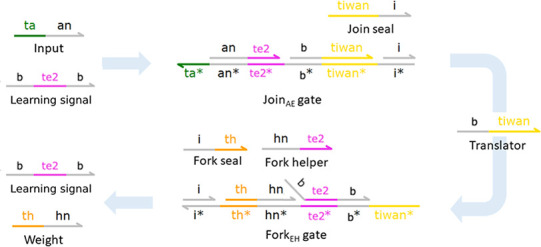
Weight accumulation (*m* = 2): A_*n*_ + . Catalytic reaction that realizes the weight accumulation function is a Join gate and a modified variant of the Fork gate. In this variation, the first reactant of the Join gate and the first product of the Fork gate are a three-domain species , which represents the learning signal. Initial Fork gate complex now has a long domain b branching out of the double-stranded structure. This modification is needed to ensure complementarity with the tunable activation function.

The weight accumulation function is distinguished from standard gates in that the first reactant of the Join gate, i.e., <b te2 b> representing the learning signal  and the first product of the Fork gate are both three-domain species. The initial form of the Fork gate complex has a long domain b branching out of the double-stranded structure (Figure 13). This modification is necessary in order to allow for  to catalyze the reaction.

An alternative way to implement this mechanism could be the use of a multistep cascade of gates. This approach, however, would necessitate the use of additional toehold definitions, thus limiting the number of input channels that could be simulated.

#### Computational Complexity

Extending the d-CN to accommodate additional input channels requires the user to define a single new toehold domain definition tiwan, which is responsible for weight accumulation in each of the *N* channels. Moreover, there are six toehold domains that remain the same regardless of the number of input channels (ta, th, tb, tfsi, tism, tisi). Therefore, the system with *N* = 3 input channels requires 9 toehold definitions (6 + *N*). In addition, depending on the length of the polymer which facilitates the activation function there are at least two additional toehold domains: te0 and te1. We base the recognition of the inputs as well as other two-domain strands in the system on the long domains. There are two long domains which remain the same regardless of the number of channels (b, i) and three which need to be defined when adding another input channel (an, hn, fsin). Therefore, the system with *N* = 3 input channels requires 11 long domain definitions (2 + 3*N*).

#### Simulating the d-CN

When simulating the d-CN, we initialize the system with different amounts of gate complexes and helper strands needed for the computation by both Join and Fork gates depending on their function. Signal modulation fuel molecules are initiated at 25 000 μM, signal integration at 50 000 μM, and weight accumulation at 10 000 μM. We also initialize the fuel molecules necessary for the signal integration mechanism *Fsi*_*n*_ with 50 000 μM. Lastly, in all of the experiments, we choose to set the bolus size, i.e., the amount of *A*_*n*_ species injected to the system at each spike, to β = 10 μM. In order to model decay of *H*_*n*_ species, we introduce garbage collection molecules {th^*}[hn], which sequester and inactivate the molecular species *H*_*n*_. We inject 12 and 0.1 μM of these species to the system periodically every 1000 s.

We have been careful to use strand displacement reaction rates that are within the range that has been measured experimentally.^47^ In order to reproduce the desired dynamical behaviors, the binding rates associated with the ta, th toeholds have been set to lower values than the other toeholds; see Table S3 for details on the parameters.

**Figure 14:**
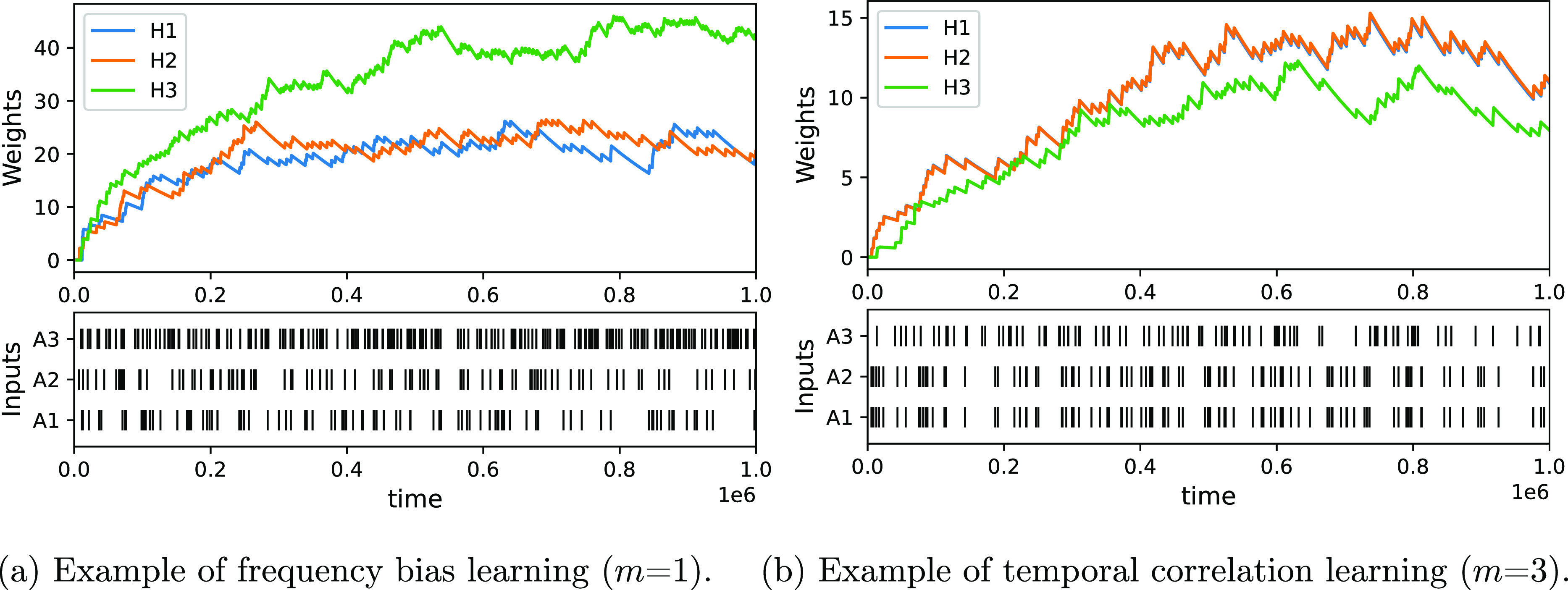
Examples of learning episodes in d-CN for (a) frequency bias task and (b) temporal correlation task. For statistical data about the weight distributions obtained over multiple runs, see Figure S2.

To determine whether the d-CN is capable of learning, we carried out a range of simulations using Visual DSD, Figure 14. We found that both infinite and detailed mode compilation could produce the intended dynamical behaviors. Similarly, we found that these behaviors could be produced in both simulations at low copy numbers (using Gillespie’s stochastic simulation algorithm) and in the fluid limit (deterministic rate equations). Accordingly, we show infinite mode deterministic simulations in the main article and other simulations in the SI (Figure S3).

**Figure 15:**
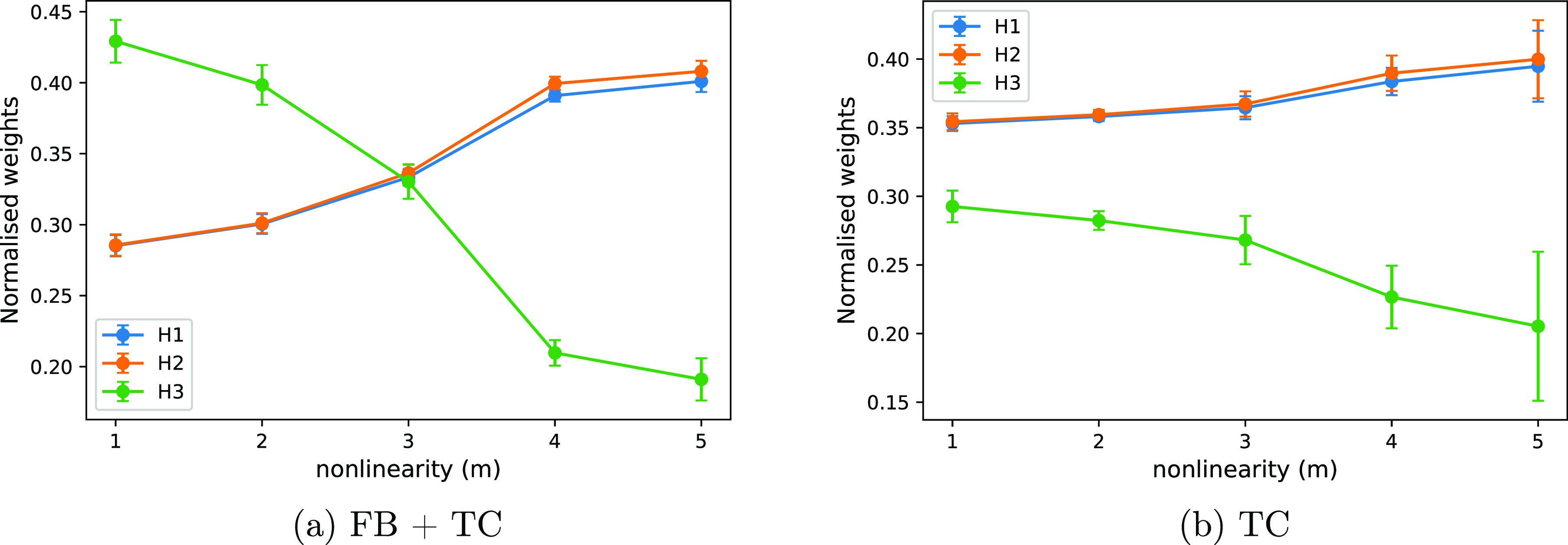
Normalized steady state weights as a function of the length of the activation function polymer. As the polymer is extended, the activation function becomes steeper and therefore requires a correlation of at least two signals to trigger learning. Therefore, the DNA neuron becomes better at recognizing temporal correlations when *m* is high.

To check whether the d-CN behaves as expected, we test its ability to distinguish the two types of biases on tasks where *A*_2_ is temporally correlated with *A*_1_ and further analyze how this depends on the nonlinearity/polymer length (Figure 15). First, we consider a scenario where *A*_3_ is both uncorrelated with *A*_1_/*A*_2_ and has a spiking frequency twice as high as the other input channels (0.0002 Hz; Figure 15a). Consistent with the CN, the d-CN is sensitive to frequency bias when the nonlinearity is low, corresponding to the weights of *A*_3_ being high for *m* = 1. Vice versa, in the case of high nonlinearity, the d-CN recognizes the temporal correlations, corresponding to the weights of *A*_1_ and *A*_2_ being high. When removing the frequency bias of *A*_3_, the system still differentiates between uncorrelated and correlated inputs but the ability to distinguish the two types of signals increases with *m* (see Figure 15b).

**Figure 16:**
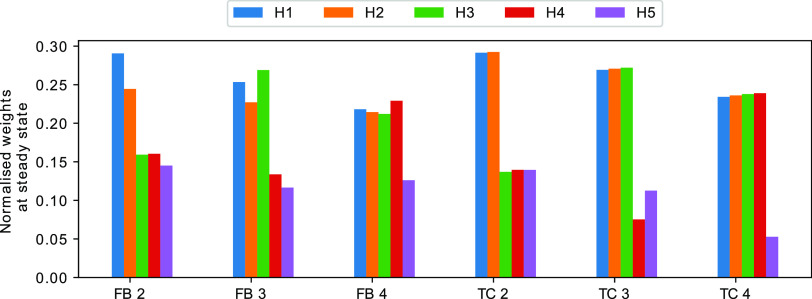
Same as Figure 4 but for the d-CN. Data was only collected after the weights reached the steady state (after 800 000 time units).

We also compared the ability of the d-CN directly with the CN. We found that the d-CN is able to detect both FB and TC biases (Figure 16). However, in the TC task the indication of the temporal order of the input signals is subtle in the sense that the steady state weights of the correlated channels are almost the same with only a slight difference indicating temporal order.

## Discussion

To the best of our knowledge, the CN is the first fully autonomous chemical model of a Hebbian spiking neuron. While it is unlikely that the basic model can be engineered as is, it has some features that make the system interesting from a fundamental point of view.

One of the attractive features of the (basic) CN neuron is that it is microreversible and therefore thermodynamically plausible. This makes it a useful theoretical tool to probe the thermodynamics of learning. While a thorough analysis of the energy requirements of the system is beyond the scope of this article, we note that the physical plausibility of the model has highlighted resource requirements of computation. In particular, we found that increasing the nonlinearity comes at an additional cost in resources. The CN suffers from starvation of *B* molecules as *m* increases. For a sufficiently high number of *m*, this leads to a breakdown of the mechanisms and the system loses its ability to detect coincidences, as illustrated in Figure 7. This “starvation” effect can be alleviated by increasing the bolus size (while keeping the threshold fixed; Figure 7). In a biological context, the increase of the bolus size comes at a direct synthesis cost if the molecules that make up the bolus need to be made by the cell. Yet, even if we assume that the particles are, somehow, pre-existing, injecting a bolus requires chemical work, which is proportional to the number of particles, i.e., the bolus size. Hence, there is a fundamental thermodynamic cost involved in computing the nonlinearity. We are not aware of any formal proofs that show that computing nonlinearities necessarily requires an increased energy requirement. It therefore remains an open question whether or not this is a feature of the particular model choices or the manifestation of a deeper constraint. Interestingly, the FB task, which does not rely on nonlinearities, can be solved with much simpler and thermodynamically cheaper designs, e.g., a simple decaying particle.

While the basic CN does not lend itself to a direct implementation in biochemistry, we presented a compartmentalized interpretation of the system that is biologically more plausible. It interprets different input species and indeed the internal state molecule *B* as one and the same species but contained in different compartments. This makes the system feasible, in principle. Although creating many compartments with the required dynamics may remain challenging, significant progress has been made in recent years toward programming molecular systems in protocells.^48^

Interestingly, there are structural similarities between the c-CN and the *lac* system in *E. coli*.^49^ The essence of the *lac* system is that it only switches on the lactose metabolism (the equivalent to the weight molecules in the compartment) when it is stimulated by lactose in the environment (i.e., *B*). The principle of operation of the *lac* system is similar to that of the c-CN, except that *E. coli* does of course not export lactose to the environment. Taking this analogy seriously, it would be interesting to consider whether catabolite repression, which is a moderately complex decision process, can be mapped to a simple neural network.

Among the three versions of the chemical neuron that we presented, we found that all could reproduce the same qualitative behaviors (Figures 3 and S9). However, given that all three of them are different designs, each version required its own parametrization, which had to be found by manual exploration in each case. It is thus not possible to reproduce the behavior of one model with another one exactly. Qualitatively, however, we found the same behaviors in all models. The only major difference was on the TC task. Unlike the other two versions of the chemical neuron, the d-CN did not clearly highlight the temporal order of input signals (Figure 16). While the d-CN indicates a strong difference between the correlated and the noncorrelated species, the weight difference between the correlated channels which should indicate the temporal order is marginal. Whether this can be improved with a better parametrization or whether this points to a fundamental limitation of the model must remain an open question.

From an engineering perspective, the d-CN is certainly the easiest to realize experimentally. DNA circuits are much less prone to crosstalk than more standard biochemical reaction networks. Synthesizing DNA molecules is now a routine procedure. There are, however, several elements of the d-CN design that will require careful consideration before an implementation can be done. For any practical use, one would need to interface the DNA computer with the in vivo target systems. How to do this in a general way remains an open question, but there have been a number of previous systems that indicate possible pathways.^50−53^

More specifically, for the d-CN, there are a number of experimental challenges that need to be addressed. In order to ensure that the kinetics of the d-CN are conserved throughout the learning and testing phases, we require the activation species *B* to decay. To remove the *B* species, we employ simple helper complexes, which are periodically replenished during the simulation. These complexes are capable of making *B* and *H*_*n*_ species unreactive, thereby removing them from the system. In order to achieve better reproducibility of the results, the experimental realization of this would necessitate a relatively frequent or continuous supply of these DNA complexes. While difficult to achieve experimentally, there are known techniques to overcome the need for frequent replenishment, including the use of buffered gates^40^ or timer circuits.^54^

Scaling the system to more input channels requires additional short domain sequences per new channel. Prima facia the scaling up of the d-CN is therefore limited by the availability of orthogonal short domain sequences. A redesign based on localized design principles could be a feasible solution if the number of toeholds becomes a problem. Here, instead of using a different set of long and short domains, distinct channels could be implemented through physical separation of the species.^55^

Describing the model as a neuron encourages the question of building networks capable of complex computational tasks. A major impediment for building networks of d-CN could be the immediate injection of *A* species to the neurons in the next layers of the network. This would necessitate inclusion of a different activation function or a mechanism which would allow for signal propagation. Incorporating a buffered gate design^40^ could allow for a programmed release of a certain number of input species once the activation signal is produced. Nevertheless, we leave the question of constructing functional neural networks in DNA for future research.

## Supporting Information Available

Detailed description of the reaction rate constants necessary to realize CN and c-CN; lists of nucleotide sequences and binding rates for d-CN; weight distributions for FB 2 and TC 2 tasks as a function of *m*; statistical data about the weight distributions for d-CN; examples of d-CN training in other simulation modes and relearning of input statistics; study of signal modulation mechanism in the d-CN; examination of strategies for garbage collection and stability of the learnt solutions as a function of bolus size and abundance of gate molecules; analysis of performance of the CN and d-CN models on the FB and TC task, and Visual DSD code for d-CN (PDF)
