# Supplementary material for: Programming Molecular Systems To Emulate a Learning Spiking Neuron
Source: ACS Synth Biol. 2022 May 27;11(6):2055–69. doi: 10.1021/acssynbio.1c00625 (PMC9208023; doi:10.1021/acssynbio.1c00625)
Supplement: Supplementary file 1 — sb1c00625_si_001.pdf [file sb1c00625_si_001.pdf]

Supporting Information: Programming molecular systems to emulate a learning spiking neuron

Jakub Fil,[*,†] Neil Dalchau,[‡] and Dominique Chu[¶]

†*APT Group, School of Computer Science, The University of Manchester, Manchester, M13 9PL, UK*

‡*Microsoft Research, Cambridge, CB1 2FB, UK*

¶*CEMS, School of Computing, University of Kent, Canterbury, CT2 7NF, UK*

E-mail: jakub.fil@manchester.ac.uk

Supplementary Tables

CN parameters

Table S1: List of reaction rate constants in the CN model (s^{-1}).

Function	Parameters
Input	$k_{IA} = 10$, $k_{AI} = 0.000001$<br>$k_{AB} = 0.1$, $k_{BA} = 0.000001$
Activation function	$k^{+} = 1$, $k^{-} = 5$<br>$k^{-}_{last} = 0.5$
Learning	$k_{AE} = 0.05$, $k_{EA} = 0.000001$, $k_{EH} = 100$, $k_{HE} = 0.000001$<br>$k_{AH} = 0.001$, $k_{HA} = 0.000001$, $k_{HB} = 100$, $k_{BH} = 0.000001$
Leak	$k_{H\varnothing} = 0.0003$<br>$k_{B\varnothing} = 0.1$

c-CN parameters

Table S2: List of reaction rate constants in a c-CN model (s^{-1}).

Function	Reaction rates
Input	$k_{IA} = 10$, $k_{AI} = 0.000001$<br>$k_{AH} = 0.03$, $k_{HA} = 0.000001$, $k_{HB} = 100$, $k_{BH} = 0.000001$
Activation function	$k^{+} = 1$, $k^{-} = 5$<br>$k^{-}_{last} = 0.5$
Weight accumulation	$k_{AE} = 0.2$, $k_{EA} = 0.000001$, $k_{A*E} = 0.2$, $k_{EA*} = 0.000001$,<br>$k_{A*A} = 0.05$, $k_{AA*} = 0.000001$<br>$k_{A*h} = 1$, $k_{hA*} = 0.1$<br>$k_{\mathrm{leak}} = 0.0001$<br>$k_{h} = 1$
Leak	$k_{H\varnothing} = 0.0003$<br>$k_{B\varnothing} = 0.1$

List of nucleotide sequences and binding rates for d-CN

Table S3: List of toehold domains and their respective binding and unbinding rates in μM s^{-1} for the simulations carried out in the infinite compilation mode.

Signal Species	Toehold	Bind	Unbind
A_n	`ta`	1	10
H_n	`th`	0.001	10
B	`tb`	1	10
E_m	`tem`	5	10
Fsi	`tfsi`	1	10
Faf	`tfaf`	1	10
Ism	`tism`	1	10
Iaf	`tiaf`	1	10
Isi	`tisi`	1	10
Iwa_n	`itwan`	1	10

Table S4: List of the two-domain DNA strands and their respective nucleotide sequences in the d-CN activation function.

Strand	Sequence
`<ta^ a1>`	$GACA + CCCTAAACTTATCTAAACAT$
`<ta^ a2>`	$GACA + CCCATTTCAAATCAAAACTT$
`<ta^ a3>`	$GACA + CCCATTACTAATCAATTCAA$
`<th^ h1>`	$CTCAG + CCCTTTTCTAAACTAAACAA$
`<th^ h2>`	$CTCAG + CCCTTATCATATCAATACAA$
`<th^ h3>`	$CTCAG + CCCTTAACTTAACAAATCTA$
`<tb^ b>`	$ACTACAC + CCCAAAACAAAACAAAACAA$
`<te0^ b>`	$CATCG + CCCAAAACAAAACAAAACAA$
`<te1^ b>`	$TACCAA + CCCTTATCATATCAATACAA$
`<te2^ b>`	$GTCA + CCCTTATCATATCAATACAA$
`<te3^ b>`	$GCTA + CCCTTATCATATCAATACAA$
`<te4^ b>`	$TATTCC + CCCTTATCATATCAATACAA$
`<te5^ b>`	$CACACA + CCCTTATCATATCAATACAA$
`<tfsi^ fsi1>`	$ACCT + CCCTATTCAATTCAAATCAA$
`<tfsi^ fsi2>`	$ACCT + CCCTATACTATACAATACTA$
`<tfsi^ fsi3>`	$ACCT + CCCTAATCTAATCATAACTA$
`<tisi^>`	$TAGCCA$
`<tism^>`	$CCCT$
`<tiwa1^>`	$CTCAATC$
`<tiwa2^>`	$CCTACG$
`<tiwa3^>`	$TCTCCA$
`<i>`	$CCCTTTACATTACATAACAA$

Supplementary Figures

Weight distributions for FB 2 and TC 2 tasks.

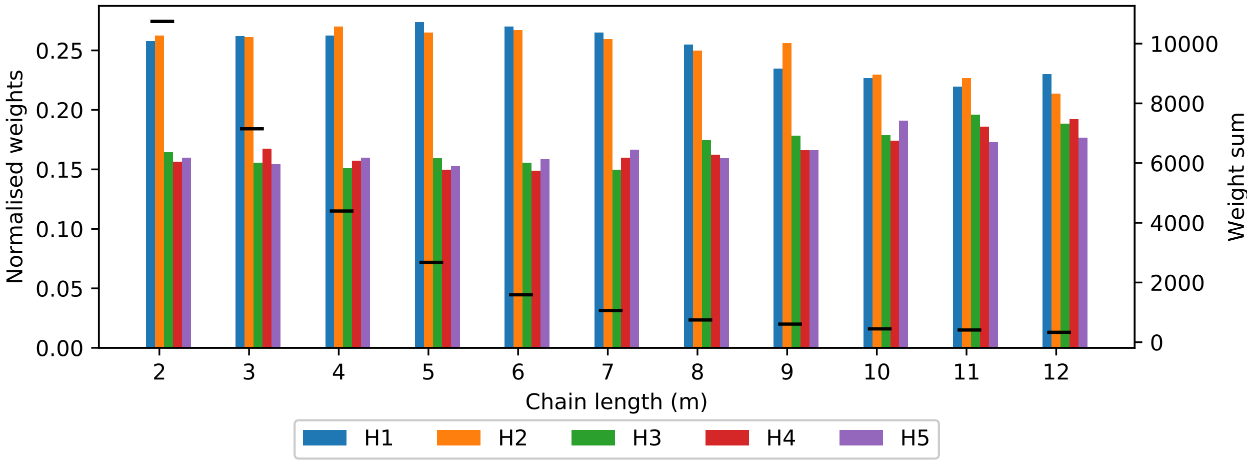

(a) FB 2

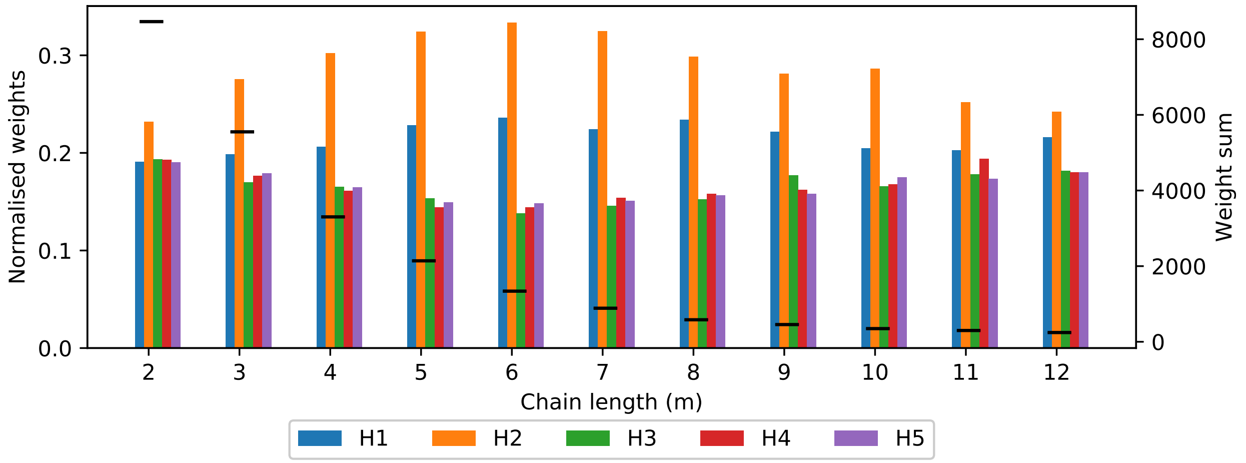

(b) TC 2

Figure S1: Normalised weights for (a) FB 2 and (b) TC 2 tasks as a function of chain length m. The y-axis on the right side of the figures describes the sum of weights across all input channels, which is a normalisation constant for the corresponding sets of weights. It can be noticed that the accumulation of weights becomes more difficult as the chain length increases. However, it's worth noting that both low and high m result in low variance of the weight representations.

Examples of learning episodes

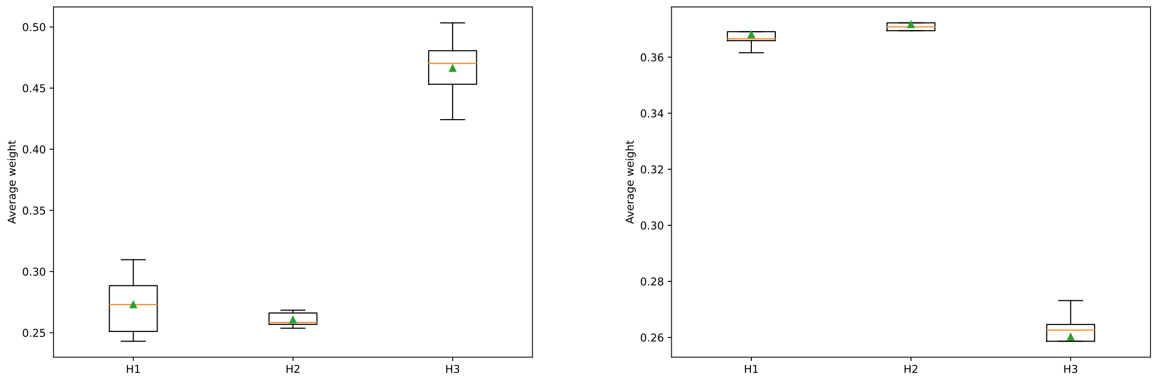

(a) Average weights for frequency bias ($m = 3$). (b) Average weights for temporal correlation ($m = 1$).

Figure S2: Normalised weights in the steady state for input data with (a) frequency bias, and (b) temporal correlation. The values represent averages over 200000s of stimulation, and were only collected once the system reached a homeostatic state (after 800000s). We then repeat each experiment 10 times, and calculate the statistics. We define the temporal correlation as pairing of inputs from channels A_1 and A_2. The inputs from A_1 always precede that of the A_2 with a fixed temporal distance δ. We can see that the steady state abundances of channel specific H_n molecules reflect the temporal order of the inputs provided. Moreover, the input channel that spiked in an asynchronous way (A_3) accumulated lower weights than the temporally correlated channels. In the frequency bias experiment we assume A_1 to come at frequency twice as high as that of the two other input channels. The system learns by accumulating steady state abundances of weight molecules H_n for each channel. After training, the weights of the channels with a high input frequency will be high, whereas the ones less likely to spike are on a similar low level.

Simulation of learning tasks

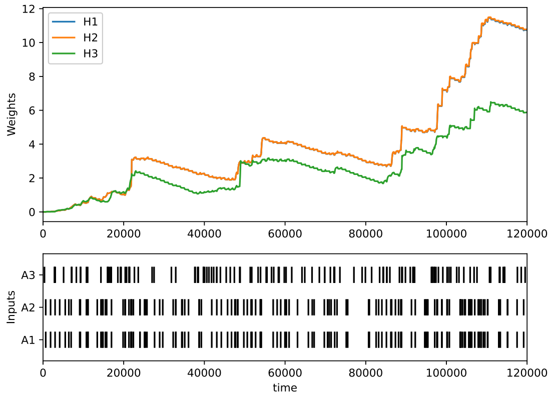

(a) Example of temporal correlation learning.

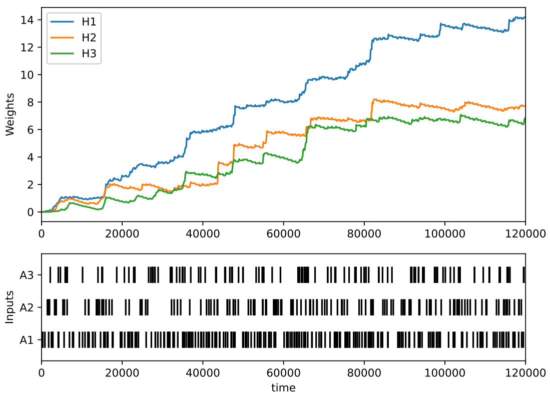

(b) Example of frequency bias learning.

Figure S3: Example learning episode for (a) temporal correlation task, and (b) frequency bias task simulated using a detailed compilation mode of Visual DSD. The detailed mode is the most realistic setting, which assumes that binding, unbinding and branch migration have finite rates.

Learning new input statistics

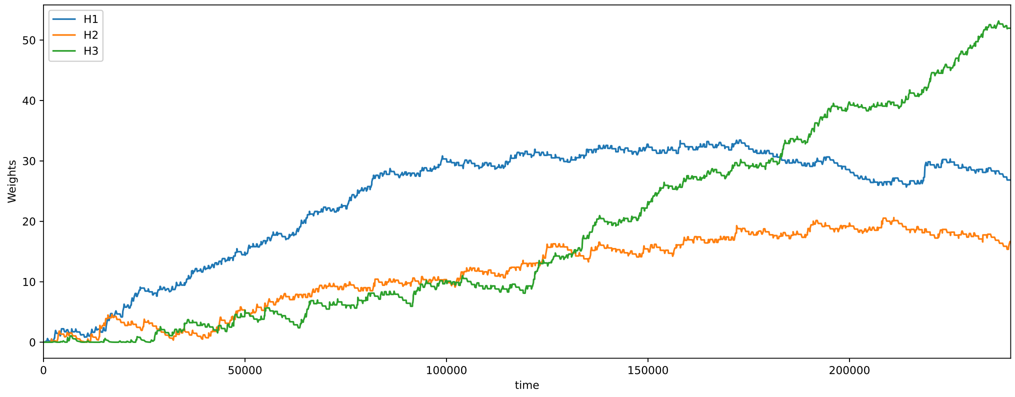

(a) Example of frequency bias learning with input statistics changing during the simulation

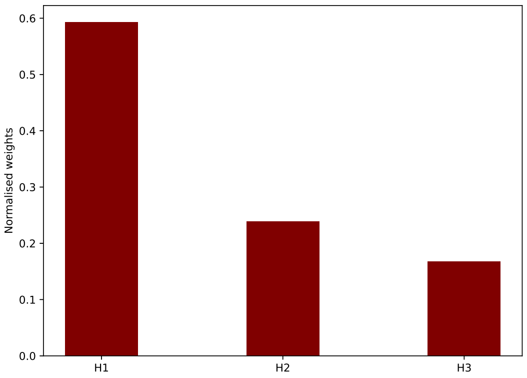

(b) Normalised weights at t=120000.

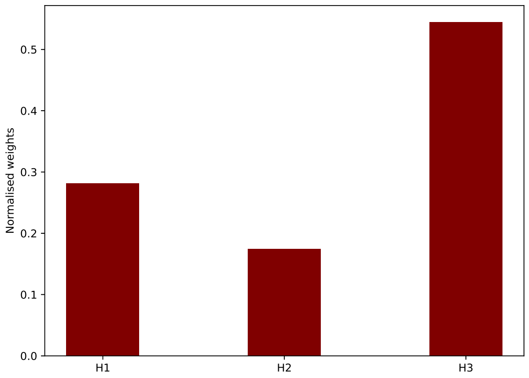

(c) Normalised weights at t=240000.

Figure S4: We examine a case when the statistical bias of the inputs changes during the simulation. The simulation starts with inputs having a statistical bias towards inputs from channel A1, which arrive twice as frequently compared to the other channels. At t=120000 the statistics change and A3 becomes the statistically over-represented input channel. The neuron's weights reflect this change and the weight associated with the new biased input channel increases.

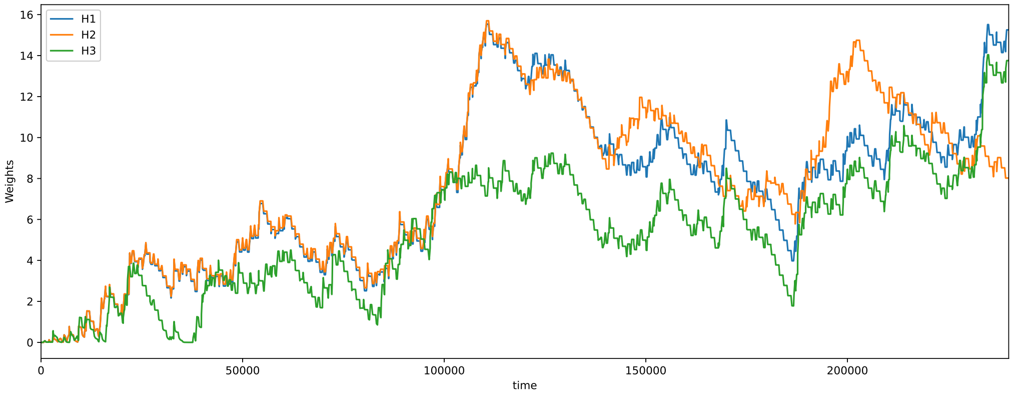

(a) Example of temporal correlation learning with input statistics changing during the simulation

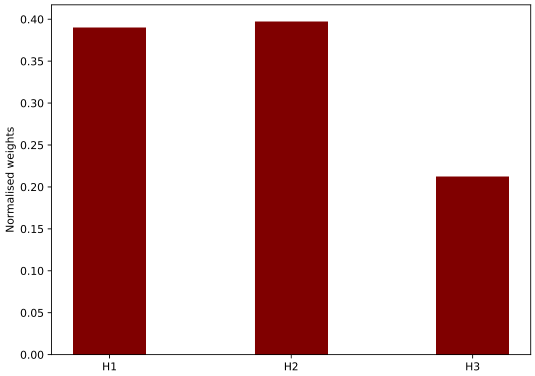

(b) Normalised weights at t=120000.

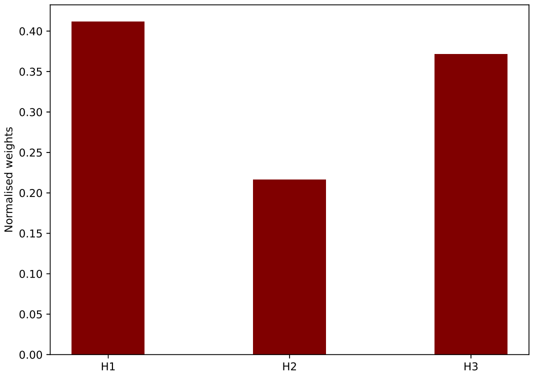

(c) Normalised weights at t=240000.

Figure S5: We examine a case when the statistical bias of the inputs changes during the simulation. The simulation starts with inputs having a temporal correlation between inputs A1 and A2, where the inputs from A2 always arrive 1s after that of A1. After 120000s, the weight associated with the second input channel H2 is slightly higher than H1, and both of them are significantly higher than H3. At t=120000 the statistics change and we introduce a correlation between A1 and A3, where the inputs from A1 always arrive 1s after that of A3. The neuron's weights reflect this change at t=240000. Now, the weight associated with the first input channel H1 is slightly higher than H3, and both of them are significantly higher than H2.

Signal modulation in the DNA neuron

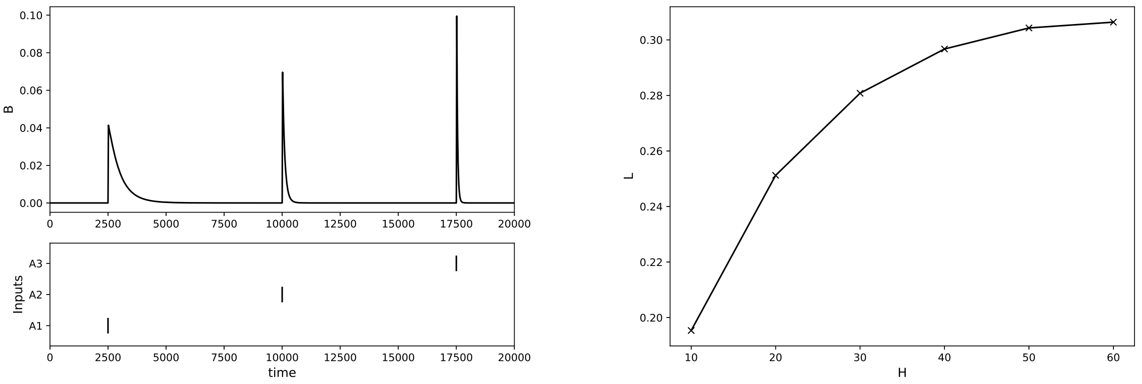

(a) Internal state (B) as a function of H_n.

(b) Learning signal ($\mathcal{E}$) as a function of H_n.

Figure S6: Signal modulation in d-CN ($m = 1$). (a) We inject 5 μM of A_n molecules to the system at $t = 100$, 300, and 500. Each channel has a different amount of H molecules associated with it: $H_1 = 10$, $H_2 = 30$, and $H_3 = 50$. This has the effect that the conversion of A_1 is slower than that of A_2, which is in turn slower than the conversion of A_3. (b) The influence of inputs on the concentration of L species as a function of its weight (H). The d-CN shows a different amount of activation given inputs from differently weighted channels. Here, we assume a constant decay of H_n and B species with rate constants $k_H = 0.00002$ and $k_B = 2$.

Strategies of garbage collection

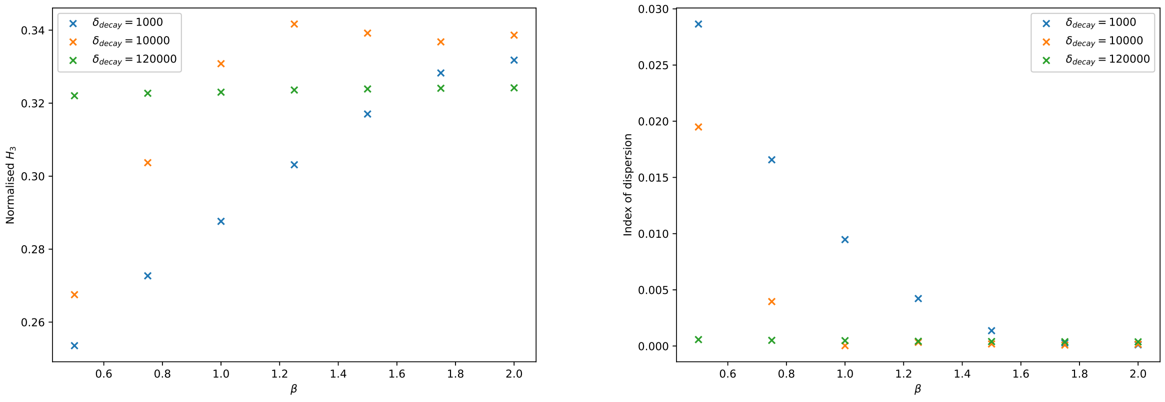

(a) Normalised H_3. (b) Index of dispersion.

Figure S7: We examine the performance of different strategies of supplying garbage collector molecules to the system as a function of bolus size β. We vary the temporal distance between subsequent injections of these species (δ_{decay}). We measure the diversity of the weight set using index of dispersion, i.e. the standard deviation divided by the mean of the weights. As expected, the more frequent but smaller injections (more similar to a decay with a constant rate) are more conducive to learning. The extreme case of $\delta_{\text{decay}} = 120000$ demonstrates that the system fails to learn if the garbage collection complexes are provided only once at the beginning of the simulation.

Stability of the d-CN

Here, we investigate the ability of the systems to distinguish temporarily correlated inputs as a function of the bolus size β. Fig. S8 shows that the increase of β results in less diverse weight representations. We use the index of dispersion, i.e. the standard deviation divided by the mean of the weights, as a measure of diversity in the weight set. As the amount of A_n molecules injected at each spike increases, the system's performance declines as a result of resource starvation. Each input spike results in a complete release of E molecules, regardless of the abundance of B molecules. This results in the steady state weight of the uncorrelated input (H_3) approaching the weights of the two other inputs. As a consequence, the system is no longer able to detect temporal correlations. Moreover, we vary the abundance of gate fuel molecules available at the beginning of each simulation. As the amount of available gate

complexes increases the ability of the system to distinguish temporally correlated inputs also increases. Therefore, we show that the performance of temporal correlation detection can be increased at a cost of more fuel molecules, and therefore longer simulation time.

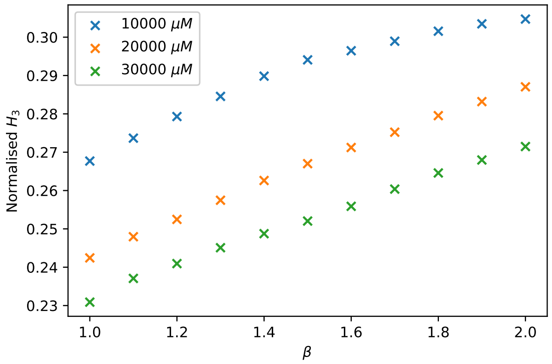

(a) Normalised H_3.

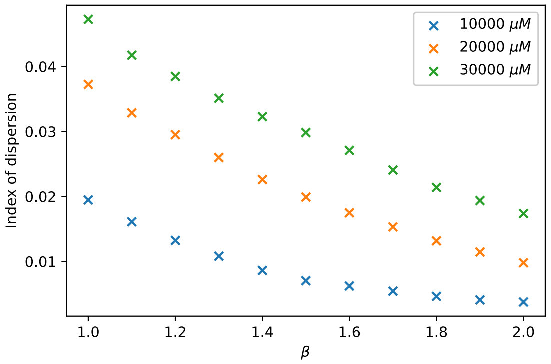

(b) Index of dispersion.

Figure S8: (a) The normalised value of the uncorrelated weight (H_3), and (b) index of dispersion of the weights at the steady state as a function of the bolus size β. The index of dispersion is a measure of diversity of the steady state weights, hence indicates how well the d-CN distinguished between input channels. This means that an input stream with no bias would result in an index of dispersion ≈ 0.

Performance of the CN and d-CN on the FB and TC task

When using the CN to detect a FB, the difference between the steady state weight abundances will reflect the difference of the frequencies with which the input channels fired, although the exact relationship between the two is not immediately clear. In order to understand this better, we considered a CN with three input channels. We then varied the frequency of channel 1 while keeping the input frequency to channel 2 fixed and recorded the ratio w_1/w_2 as a function of f_1/f_2. We found that the weight ratio was proportional to the frequency ratio (Fig. S9a). While it remains unclear to what extent this qualitative result generalises to more complicated cases, it is apparent that CN is able to detect very small

biases, albeit with a correspondingly small output signal strength.

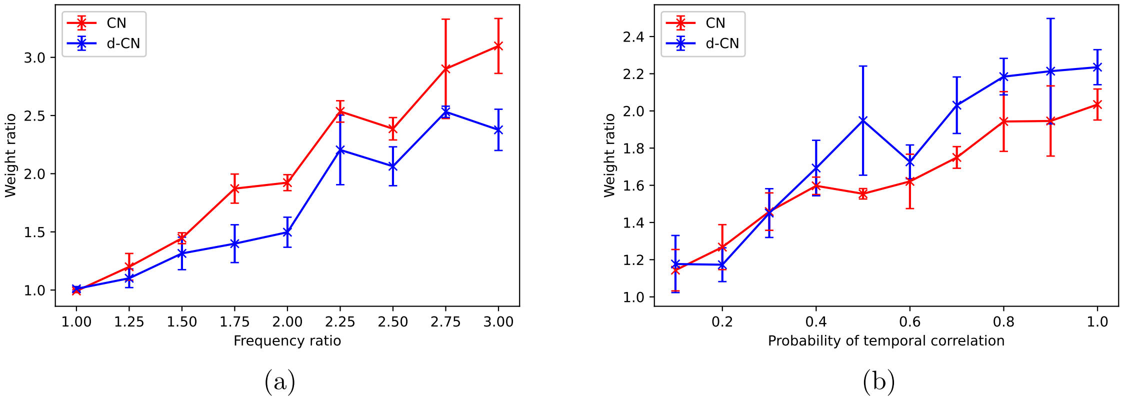

Figure S9: The response of the neuron, as measured by the ratio of the first and the second channel weight, as a function of the signal strength. (a) We kept the frequency of the first channel fixed at 1 Hz and decreased the frequency of the second channel. The non-linearity was set to 1. (b) Both input channels have the same frequency of 1 Hz, but we varied the probability that an output spike of the second channel follows an input to channel 1. The nonlinearity was set to 5.

We performed an equivalent analysis with the TC task. We varied the probability of an input spike in channel 1 being followed by an input spike in channel 2, while keeping the total frequency of all input channels constant. So, for example, a probability of 0.5 means that on average every second input spike of channel 1 is followed by an input spike of channel 2 after a delay of δ and half of the input spikes of channel 2 occur at random times. Again, we find that even for small probabilities, there is a reliable difference in weights between the first and the second channel (Fig. S9b).

Visual DSD code for d-CN model

```
directive simulation {final=1000000; plots=[B(); L(); H1(); H2(); H3(); A1(); A2(); A3()]}
directive simulator deterministic
directive deterministic {stiff=true}
directive units{concentration=uM}
directive compilation infinite
directive parameters [bOUT=2; Ein=5; AFgateIn=10; backIn=10; rateb=1; tedeg=1; t1b_deg =0.1; NovelB1=10; Ain = 10; SMFin = 0.5; WAFin = 0.5; SIFin = 0.5; AFFin=1; NdegE = 0; NdegH = 0.05; Hs = 0; kdegB = 0.1; kdegE = 0.1; kdegH = 0.000005]

//// Toehold domain reactivity
dom ta = {bind=1; unbind=10; colour="green"} // Inputs
dom th = {bind=0.001; unbind=10; colour="orange"} // Weights
dom tb = {bind=1; unbind=10; colour="#00fbff"} // State
dom te0 = {bind=5; unbind=10; colour="#eb34e8"} // Activation
dom te1 = {bind=5; unbind=10; colour="#eb34e8"} // Activation
dom te2 = {bind=5; unbind=10; colour="#eb34e8"} // Activation
dom te3 = {bind=5; unbind=10; colour="#eb34e8"} // Activation
dom te4 = {bind=5; unbind=10; colour="#eb34e8"} // Activation
dom te5 = {bind=5; unbind=10; colour="#eb34e8"} // Activation
dom tfsi = {bind=1; unbind=10; colour="black"} // Fuel for SI

//// Translator toeholds reactivity
dom tiwa1 = {bind=1; unbind=10; colour="#ffe000"} // WA1
dom tiwa2 = {bind=1; unbind=10; colour="#ffe000"} // WA2
dom tiwa3 = {bind=1; unbind=10; colour="#ffe000"} // WA3
```

```
dom tiwa4 = {bind=1; unbind=10; colour="#ffe000"} // WA4
dom tism = {bind=1; unbind=10; colour="blue"} // SM
dom tisi = {bind=1; unbind=10; colour="red"} // SI

//// Activation
def E0() = <te0^ b>
def E1() = <te1^ b>
def E2() = <te2^ b>
def E3() = <te3^ b>
def E4() = <te4^ b>

//def L() = <b te1^ b> // Learning signal (m=1)
//def L() = <b te2^ b> // Learning signal (m=2)
//def L() = <b te3^ b> // Learning signal (m=3)
//def L() = <b te4^ b> // Learning signal (m=4)
def L() = <b te5^ b>  // Learning signal (m=5)

def nE0() = <b te0^>
def nE1() = <b te1^>
def nE2() = <b te2^>
def nE3() = <b te3^>
def nE4() = <b te4^>

def nB() = <b tb^>

//// Inputs
def A1() = <ta^ a1>
def A2() = <ta^ a2>
def A3() = <ta^ a3>
```

```
def A4() = <ta^ a4>

//// Weights
def H1() = <th^ h1>
def H2() = <th^ h2>
def H3() = <th^ h3>
def H4() = <th^ h4>

//// State
def B() = <tb^ b>

//// Fuels
//// Join gate (R1 + R2 <-> T)
def Join(ta, a, tb, b, tr) = {ta^*}[a tb^]:[b tr^]:[i]
//// Fork gate (T <-> P1 + P2)
def Fork(ta, a, tb, b, tr) = [i]:[ta^ a]:[tb^ b]{tr^*}
def Fork_WA(ta, a, tb, b, tr) = [i]:[ta^ a]:<b>[tb^ b]{tr^*}

//// Decay modules
def degE() = {te^*}[e]                          // E removal
def degB() = {tb^*}[b]                          // B removal
def degH1() = {th^*}[h1]                        // H1 removal
def degH2() = {th^*}[h2]                        // H2 removal
def degH3() = {th^*}[h3]                        // H3 removal
def degH4() = {th^*}[h4]                        // H4 removal

//// Weight accumulation: An + Ex <-> Ex + Hn (OLD) mx
def WA_fuel_mx(an, hn, tiwan, fuel, time) =
( fuel Join(ta, an, te5, b, tiwan) @ time
```

```
| fuel Fork_WA(th, hn, te5, b, tiwan) @ time
| fuel <hn te5^> @ time
| fuel <i th^> @ time
| fuel <tiwan^ i> @ time
)

//// Signal modulation: An + Hn <-> Hn + B
def SM_fuel(an, hn, fuel, time) =
( fuel Join(ta, an, th, hn, tism) @ time
| fuel Fork(tb, b, th, hn, tism) @ time
| fuel <b th^> @ time
| fuel <i tb^> @ time
| fuel <tism^ i> @ time
)

//// Signal integration: An + In <-> In + B - NEW
def SI_fuel_new(an, in, fuel, time) =
( fuel Join(ta, an, tfsi, in, tisi) @ time
| fuel Fork(tb, b, tfsi, in, tisi) @ time
| fuel <b tfsi^> @ time
| fuel <tfsi^ in> @ time
| fuel <i tb^> @ time
| fuel <tisi^ i> @ time
)

//// Signal integration: I + An <-> An + B - OLD
def SI_fuel_old(in, an, fuel, time) =
( fuel Join(tfsi, in, ta, an, tisi) @ time
| fuel Fork(tb, b, ta, an, tisi) @ time
```

```
| fuel <b ta^> @ time
| fuel <tfsi^ in> @ time
| fuel <i tb^> @ time
| fuel <tisi^ i> @ time
)

//// Activation function: B + Faf <-> Faf + E
def AF_newE_5(fuel_gate, time) =
( fuel_gate {tb^*}[b te0^]:[b tb^]:[b te1^]:[b tb^]:[b te2^]:[b tb^]:[b te3^]:[b
    tb^]:[b te4^]:[b te5^]<b> @ time
)

//// Activation function: B + Faf <-> Faf + E
def AF_newE_4(fuel_gate, time) =
( fuel_gate {tb^*}[b te0^]:[b tb^]:[b te1^]:[b tb^]:[b te2^]:[b tb^]:[b te3^]:[b
    te4^]<b> @ time
)

//// Activation function: B + Faf <-> Faf + E
def AF_newE_3(fuel_gate, time) =
( fuel_gate {tb^*}[b te0^]:[b tb^]:[b te1^]:[b tb^]:[b te2^]:[b te3^]<b> @ time
)

//// Activation function: B + Faf <-> Faf + E
def AF_newE_2(fuel_gate, time) =
( fuel_gate {tb^*}[b te0^]:[b tb^]:[b te1^]:[b te2^]<b> @ time
)

//// Activation function: B + Faf <-> Faf + E
```

```
def AF_newE_1(fuel_gate, time) =
( fuel_gate {tb^*}[b te0^]:[b te1^]<b> @ time
)

//// Short example of randomly generated input with frequency bias
( 0 B() | B() ->{bOUT}
| 0 H1() | H1() ->{kdegH}
| 0 H2() | H2() ->{kdegH}
| 0 H3() | H3() ->{kdegH}

| Ein E0()
| Ein E1()
| Ein E2()
| Ein E3()
| Ein E4()

| backIn nE0()
| backIn nE1()
| backIn nE2()
| backIn nE3()
| backIn nE4()

| backIn nB()
| 0 L()
| AF_newE_5(AFgateIn, 0)

| SM_fuel(a1, h1, 25000, 0)
| SI_fuel_old(i1, a1, 80000, 0)
| WA_fuel_mx(a1, h1, tiwa1, 10000, 0)
```

```
| SM_fuel(a2, h2, 25000, 0)
| SI_fuel_old(i2, a2, 80000, 0)
| WA_fuel_mx(a2, h2, tiwa2, 10000, 0)

| SM_fuel(a3, h3, 25000, 0)
| SI_fuel_old(i3, a3, 80000, 0)
| WA_fuel_mx(a3, h3, tiwa3, 10000, 0)

| Ain A1() @ 1376
| Ain A1() @ 1794
| Ain A3() @ 5713
| Ain A3() @ 5963
| Ain A1() @ 9357
// etc.
)
```
